# Antimicrobial resistance and clonality of *Staphylococcus aureus* causing bacteraemia in children admitted to the Manhiça District Hospital, Mozambique, over two decades

**DOI:** 10.3389/fmicb.2023.1208131

**Published:** 2023-07-24

**Authors:** Marcelino Garrine, Sofia Santos Costa, Augusto Messa, Sérgio Massora, Delfino Vubil, Sozinho Ácacio, Tacilta Nhampossa, Quique Bassat, Inacio Mandomando, Isabel Couto

**Affiliations:** ^1^Centro de Investigação em Saúde de Manhiça (CISM), Maputo, Mozambique; ^2^Global Health and Tropical Medicine, GHTM, Instituto de Higiene e Medicina Tropical, IHMT, Universidade Nova de Lisboa, UNL, Lisbon, Portugal; ^3^Instituto Nacional de Saúde (INS), Ministério da Saúde, Maputo, Mozambique; ^4^ISGlobal, Hospital Clínic-Universitat de Barcelona, Barcelona, Spain; ^5^ICREA, Barcelona, Spain; ^6^Department of Pediatrics, Hospital Sant Joan de Déu, Universitat de Barcelona, Esplugues, Barcelona, Spain; ^7^CIBER de Epidemiología y Salud Pública, Instituto de Salud Carlos III, Madrid, Spain

**Keywords:** paediatric, bacteraemia, *Staphylococcus aureus*, MRSA, MDR, *spa* typing, MLST, Mozambique

## Abstract

**Background:**

*Staphylococcus aureus* is one of the main causes of bacteraemia, associated with high mortality, mainly due to the occurrence of multidrug resistant (MDR) strains. Data on antibiotic susceptibility and genetic lineages of bacteraemic *S. aureus* are still scarce in Mozambique. The study aims to describe the antibiotic susceptibility and clonality of *S. aureus* isolated from blood cultures of children admitted to the Manhiça District Hospital over two decades (2001–2019).

**Methods:**

A total of 336 *S. aureus* isolates detected in blood cultures of children aged <5 years were analyzed for antibiotic susceptibility by disk diffusion or minimal inhibitory concentration, and for the presence of resistance determinants by PCR. The clonality was evaluated by S*ma*I-PFGE, *spa* typing, and MLST. The SCC*mec* element was characterized by SCC*mec* typing.

**Results:**

Most *S. aureus* (94%, 317/336) were resistant to at least one class of antibiotics, and one quarter (25%) showed a MDR phenotype. High rates of resistance were detected to penicillin (90%) and tetracycline (48%); followed by erythromycin/clindamycin (25%/23%), and co-trimoxazole (11%), while resistance to methicillin (MRSA strains) or gentamicin was less frequent (≤5%). The phenotypic resistance to distinct antibiotics correlated well with the corresponding resistance determinants (Cohen’s *κ* test: 0.7–1.0). Molecular typing revealed highly diverse clones with predominance of CC5 (17%, 58/336) and CC8 (16%), followed by CC15 (11%) and CC1 (11%). The CC152, initially detected in 2001, re-emerged in 2010 and became predominant throughout the remaining surveillance period, while other CCs (CC1, CC5, CC8, CC15, CC25, CC80, and CC88) decreased over time. The 16 MRSA strains detected belonged to clones t064-ST612/CC8-SCC*mec*IVd (69%, 11/16), t008-ST8/CC8-SCC*mec*NT (25%, 4/16) and t5351-ST88/CC88-SCC*mec*IVa (6%, 1/16). Specific clonal lineages were associated with extended length of stay and high in-hospital mortality.

**Conclusion:**

We document the circulation of diverse MDR *S. aureus* causing paediatric bacteraemia in Manhiça district, Mozambique, requiring a prompt recognition of *S. aureus* bacteraemia by drug resistant clones to allow more targeted clinical management of patients.

## 1. Introduction

*Staphylococcus aureus* bacteraemia (SAB) is one of the most common bloodstream infections worldwide (Kern and Rieg, 2020; Bai et al., 2022). The mortality associated with SAB is higher (29%–63%) (Kaasch et al., 2014) compared to bloodstream infections caused by other Gram-positive pathogens (Gijón et al., 2016). The burden of SAB is increasing around the globe (Kern and Rieg, 2020) and the treatment of affected patients is challenged by the emergence of multidrug resistant (MDR) and methicillin-resistant *S. aureus* (MRSA) strains (Murray et al., 2022). Most antibiotic resistance exhibited by *S. aureus* is due to resistance genes encoded on the chromosome or those acquired by horizontal transfer from other *S. aureus* strains, as well as from other bacteria (Vestergaard et al., 2019). Furthermore, the global prevalence of MRSA is related to the dissemination of pandemic clones, and acquisition of the Staphylococcal chromosomal cassette *mec*—SCC*mec* element (which harbours the *mec* gene, encoding methicillin resistance) by local methicillin-susceptible *S. aureus* (MSSA) (Lee et al., 2018). These reasons lead the World Health Organization (WHO) to list MRSA as priority target to guide research, discovery and development of new antibiotics, because of its ability to rapidly develop resistance against multiple antibiotic classes hence limiting therapeutic options (Tacconelli et al., 2018).

Molecular typing studies on *S. aureus* have been using well-described methods, such as pulsed-field gel electrophoresis (PFGE) (Chung et al., 2000), multilocus sequence typing (MLST) (Enright et al., 2000), staphylococcal protein A typing (*spa* typing) (Frénay et al., 1996) and SCC*mec* typing (Zhang et al., 2005). These methods allow investigating outbreaks and long-term epidemiological studies (Strommenger et al., 2006; Mellmann et al., 2008). More recently, the introduction of WGS tools has allowed exhaustive strain characterization (Raven et al., 2019). However, detailed molecular characterization of clinical *S. aureus* from Africa has been largely neglected in the past (Schaumburg et al., 2014). Although there are recent data regarding molecular characterization of *S. aureus* originating from different African countries (Perovic et al., 2017; Ruffing et al., 2017; Amoako et al., 2019; Kyany’a et al., 2019; Mzee et al., 2021), they are still scarce compared to the ones available from other regions of the globe (Toleman et al., 2017; Baig et al., 2020; Cabrera et al., 2020), reinforcing the need for additional studies to understand the local epidemiology of this important pathogen (Schaumburg et al., 2014; Tong et al., 2015). *S. aureus* was previously reported as the third leading cause of bacteraemia among children in Mozambique (Sigaúque et al., 2009) and the first cause among newborns and infants under the age of 3 months (Sigaúque et al., 2018); yet, in-depth characterization are still lacking. Early molecular characterization of a sub-set of approximately 20% of paediatric *S. aureus* causing bacteraemia in our study community, provided a snapshot on *S. aureus* bacteraemia in our region, showing high strain diversity with predominance of the clonal complexes (CC) CC5, CC8, CC15, and CC25, and the *spa* types t064 and t084 (Vubil et al., 2017). However, one important study limitation was the small number of isolates analysed and possible bias on sample selection, which did not allow drawing robust conclusions on the potential contribution and role of MDR/MRSA impact on clinical outcome. We expanded this earlier study and found a declining rate of SAB, although the disease remains an important cause of child mortality in our study area (Taylor et al., 2020; Garrine et al., 2023), possibly in relation to the resistance to the first line of empirical treatment in use, suggesting an urgent need to review current policy recommendations (Garrine et al., 2023). Therefore, we herein aim to fill the gap of knowledge associated with paediatric SAB, by describing the antibiotic susceptibility, presence of resistance determinants and clonality of *S. aureus* causing bacteraemia among children under 5 years of age in Manhiça District, Mozambique, in the last two decades (2001–2019).

## 2. Methodology

### 2.1. Site description

The Manhiça District Hospital (MDH) is a referral health facility for Manhiça district, a rural area located 80 km North of Maputo city, Southern Mozambique. A full description of the geographical and socio-demographic characteristics of the study community has been presented and updated elsewhere (Sacoor et al., 2013; Nhacolo et al., 2021). The*“Centro de Investigação em Saúde de Manhiça”* (CISM) has a continuous health and demographic surveillance system for vital events and migrations since 1996, currently covering the entire district with an estimated population of 201,845 inhabitants in 46,441 households (Nhacolo et al., 2021).

### 2.2. Specimen collection and *Staphylococcus aureus* isolation

Since 1997, the CISM and MDH have jointly operated a 24 h morbidity surveillance, with standardized collection of clinical data for all paediatric patients (<15 years of age) and a specific microbiological surveillance based on the systematic collection of blood cultures among all admitted patients (Sigaúque et al., 2009). Specifically, as part of microbiological surveillance, a single venous blood sample (1–3 mL) for bacterial isolation is routinely collected upon hospital admission for all children aged <2 years, and for children aged between 2 and <15 years with axillary temperature ≥39°C or with signs of severe illness, as described elsewhere (Sigaúque et al., 2009). In this study we focused our analysis on children aged <5 years, as 95% of *S. aureus* were isolated from this group.

### 2.3. Antimicrobial susceptibility testing

Three hundred and thirty-six frozen *S. aureus* isolates were retrieved and tested for antimicrobial susceptibility by Kirby–Bauer disk diffusion, or determination of minimal inhibitory concentration (MIC) by *E*-test. Results were interpreted according to the Clinical Laboratory Standards Institute (CLSI) guidelines (CLSI, 2023). According to the CLSI guidelines, “not susceptibility” profiles included isolates categorized as intermediate or resistant (CLSI, 2023). Multidrug resistance was defined as not susceptibility to at least one agent in at least three unrelated classes of antibiotics (Magiorakos et al., 2012). The isolates were tested against the following antibiotics: cefoxitin (FOX, 30 μg), penicillin (PEN, 10 units), ciprofloxacin (CIP, 5 μg), chloramphenicol (CHL, 30 μg), erythromycin (ERY, 15 μg), gentamicin (GEN, 10 μg), tetracycline (TCY, 30 μg), trimethoprim/sulfamethoxazole “co-trimoxazole” (SXT, 1.25/23.75 μg), clindamycin (CLID, 2 μg), daptomycin (DAP, 0.016–256 μg/mL), linezolid (LNZ, 0.016–256 μg/mL) and vancomycin (VAN, 0.016–256 μg/mL) (Mast Group, Ltd., Merseyside, United Kingdom). The cefoxitin disk was used as a surrogate for oxacillin resistance, to screen putative MRSA. Inducible clindamycin resistance was detected by the *D*-test for all isolates resistant to erythromycin and susceptible or intermediate to clindamycin. *S. aureus* strains ATCC^®^25923^™^ and ATCC^®^29213^™^ were used as quality control for disk diffusion and E-test, respectively (CLSI, 2023).

### 2.4. Screening of resistance determinants

*S. aureus* showing a not susceptibility profile were screened for the presence of the corresponding resistance determinants by conventional PCR. Briefly, the isolates were cultivated into blood agar plates and incubated overnight at 37°C. Afterward, one colony was selected from the blood agar plate and inoculated into 5 mL of BD Tryptic Soy Broth (Becton-Dickinson, Heidelberg, Germany) followed by overnight incubation at 37°C. Upon incubation, crude DNA was extracted by the boiling method according to an established protocol (Alexopoulou et al., 2006), and screened by PCR targeting the corresponding resistance genes of interest, namely, *blaZ, mecA, tet*(K), *tet*(M), *tet*(L), *erm*(C), *erm*(A), *msr*(A), *aacA-aphD*, *dfr*(G), *dfrA*(S1), and *cat*p_C221_, using specific primers and conditions (Supplementary Table S1). The main resistance determinants encoding for resistance to penicillin (*blaZ*), cefoxitin (*mecA*) and tetracycline (*tet*(K)) were screened in the entire *S. aureus* collection. The amplification products were separated in 1.5% agarose gels stained with ethidium bromide, using the 1 Kb plus or 100 bp DNA ladder (Bio-Rad) as molecular size markers.

### 2.5. Screening of mutations in quinolone-resistance determining region of *grlA* and *gyrA* genes

The strains not susceptible to ciprofloxacin were screened for mutations in the quinolone-resistance determining region (QRDR) of *grlA* and *gyrA* genes, using specific primers and conditions (Supplementary Table S1). The amplicons were purified using the NZYGelpure kit (NZYTech, Lisbon, Portugal) and sequenced by the Sanger method at STAB-Vida (Caparica, Portugal). Sequence alignment analyses with appropriate reference sequences searched in the National Center for Biotechnology Information (NCBI, Bethesda, MD, United States) public repository database were conducted using MEGA 11 (Tamura et al., 2021) to identify mutations associated with fluoroquinolone resistance (Hooper, 1999; Jones et al., 2000).

### 2.6. Molecular typing and inference of CCs

Amplification and sequencing of the hypervariable region of the *spa* gene was carried out for the entire collection (*n* = 336) as described elsewhere (Harmsen et al., 2003). The *spa* types were assigned using the Ridom Staph Type database (Ridom GmbH, Würzburg, Germany, version 2.2.5). MLST was performed for at least two representative *S. aureus* from each *spa* type (*n* = 168), using the scheme previously described (Enright et al., 2000). Allelic profiles, sequence types (STs) and CCs were assigned using the MLST *S. aureus* database^1^. Selected *S. aureus* (*n* = 160) were analyzed by PFGE to solve discrepancies and/or to increase the discriminatory power of MLST/*spa* typing results. The isolates were compared for their genetic relatedness by *Sma*I macrorestriction, according to the protocols described elsewhere (Chung et al., 2000). The restriction patterns were analyzed using BioNumerics version 7.6 (Applied Maths NV, Sint-Martens-Latem, Belgium) with Dice coefficient (1% and 0.5% of tolerance and optimization, respectively). Groups of isolates showing at least 80% of similarity were considered to share the same profile (pulsotype), and those with similarity ≥97% were considered the same subtype (Carriço et al., 2005). PFGE patterns found in a single isolate were designated single pulsotypes. Previous studies have shown high concordance between groupings obtained by *spa* typing with the classifications obtained by MLST or PFGE (Strommenger et al., 2006; Mellmann et al., 2008). Therefore, for isolates with no CC assigned by PubMLST, we inferred the CCs based on the agreement between *spa* type, PFGE and STs/CCs and, when necessary, data obtained from the literature (Supplementary Table S2). The clonal relatedness was inferred with the PHYLOViZ online version (Francisco et al., 2012), considering the STs/CCs found in this study and all STs/CCs described for *S. aureus* in the PubMLST database until December 2022.

### 2.7. Staphylococcal cassette chromosome *mec* (SCC*mec*) typing

We performed a multiplex PCR for identification of SCC*mec* types (I, II, III, and V) and subtypes (IVa, IVb, IVc, and IVd) for the MRSA strains, using primers and conditions previously described (Zhang et al., 2005).

### 2.8. Data analysis

The statistical analyses were performed using STATA version 14.1 (StataCorp LP, College Station, Texas, United States). Categorical variables were compared using the *χ*^2^ or Fisher’s exact test when appropriate, and a *p-*value of 0.05 or lower was considered statistically significant. Age groups were categorized as neonates (≤28 days), infants (29 days–11 months), toddlers (12–23 months), and young children (24–59 months) (Ministério da Saúde-Mozambique, 2011). The antimicrobial susceptibility data were analyzed through WHONET version 19.8.6 (World Health Organization, Geneva, Switzerland). The level of agreement between antibiotic susceptibility testing among selected antibiotics (penicillin, cefoxitin and tetracycline) and the resistance determinants (*blaZ*, *mecA* and *tet*(K)) was determined by Cohen’s *κ* test using GraphPad Prism.^2^ The *κ* coefficient was interpreted as no agreement (*κ* < 0), slight agreement (*κ*: 0.00–0.20), fair agreement (*κ*: 0.21–0.40), moderate agreement (*κ*: 0.41–0.60), substantial agreement (*κ*: 0.61–0.80), and almost perfect agreement (*κ*: 0.81–1.00) (Landis and Koch, 1977). The genetic diversity of the collection was calculated, based on the *spa* types and MLST, by Simpson’s index of diversity (SID) with 95% confidence interval.^3^

### 2.9. Ethics statement

The *S. aureus* collection analyzed in this study is in the scope of the ongoing morbidity and microbiological surveillance system established as part of the CISM’s Health and Demographic Surveillance System approved by the Institutional Ethics Review Board for Health at CISM, and from the National Bioethics Committee for Health. All residents of Manhiça’s district have signed an individual informed consent to become part of the ongoing surveillance.

## 3. Results

### 3.1. Demographic characteristics

From January 01, 2001 to December 31, 2019; 50,293 children aged <5 years were admitted to the MDH, and blood cultures were collected on admission for 83% (41,891) of the patients. Bacteraemia was diagnosed in 7.6% of cases (3,197/41,891) with *S. aureus* isolated in 0.9% (394/41,891) of the blood cultures, corresponding to 12.3% (394/3,197) of bacteraemic patients. The epidemiological and clinical characteristics of these patients have been described in a separate study, including the proportion of SAB as a cause of community bacteraemia across the several age strata (Garrine et al., 2023). In that early study, the SAB incidence ranged from 322.1 to 12.5 episodes/100,000 children years at risk between 2001 and 2019 (Garrine et al., 2023). The present work describes the analysis of the 336 *S. aureus* isolates recovered from 333 children with SAB over this period, including three children presenting two morphologically distinct isolates in the same blood culture.

### 3.2. Antimicrobial resistance profile

Overall, 94% (317/336) of the *S. aureus* tested were not susceptible (resistant or intermediate phenotype) to at least one antibiotic (Table 1). More specifically, 37% (124/336) were not susceptible to one antibiotic class, 32% (108/336) to two classes and 25% (85/336) were MDR. High frequencies of resistance were observed for penicillin (90%), tetracycline (48%) and erythromycin/clindamycin (25% and 23%, respectively), while resistance to chloramphenicol or gentamicin was scarce (Table 1). We found a low frequency of MRSA (5%, 16/336), mainly among infants (8%, 7/93) and young children (7%, 4/56), followed by toddlers (5%, 4/80) and neonates (1%, 1/104). Resistance to ciprofloxacin was observed in only one MSSA, which presented a MDR profile (CIP-PEN-SXT-GEN). All isolates were susceptible to vancomycin, daptomycin and linezolid. The 317 not susceptible *S. aureus* displayed 32 resistance profiles. Among the 85 MDR strains, the most common profile was PEN-TCY-ERY-CLID (31%), followed by PEN-ERY-CLID (20%) and FOX-PEN-TCY-ERY-CLID-GEN-SXT-CHL (11%). This last profile was the most commonly observed amongst MRSA (56%) (Supplementary Table S3). The morphologically distinct *S. aureus* isolated from the same blood culture (same patient) showed distinct resistance patterns, as follows: (i) PEN *vs*. PEN-ERY-CLID; (ii) TCY *vs*. TCY-ERY-CLID, and (iii) PEN *vs*. PEN-ERY.

**Table 1:** Antimicrobial resistance rate of *Staphylococcus aureus* isolated from children aged <5 years admitted with bacteraemia (*n* = 336).

Antibiotic name	NS^a^		
	R	I	Total
	*n* (%)	*n* (%)	*n* (%)
Penicillin G	304 (90)	0	304 (90)
Cefoxitin	16 (5)	0	16 (5)
Tetracycline	156 (46)	5 (1)	161 (48)
Erythromycin	69 (21)	15 (4)	84 (25)
Clindamycin	69^b^ (21)	7 (2)	76 (23)
Co-trimoxazole	32 (10)	4 (1)	36 (11)
Chloramphenicol	15 (4)	1 (<1)	16 (5)
Gentamicin	11 (3)	4 (1)	15 (4)
Ciprofloxacin	1 (<1)	0	1 (<1)

a NS, not susceptible, includes strains with resistant (R) or intermediate (I) phenotypes.

b All clindamycin resistance phenotypes observed were inducible. All the *S. aureus* were susceptible to vancomycin, daptomycin and linezolid.

### 3.3. Patterns of antimicrobial resistance over the surveillance period

**Figure 1:**
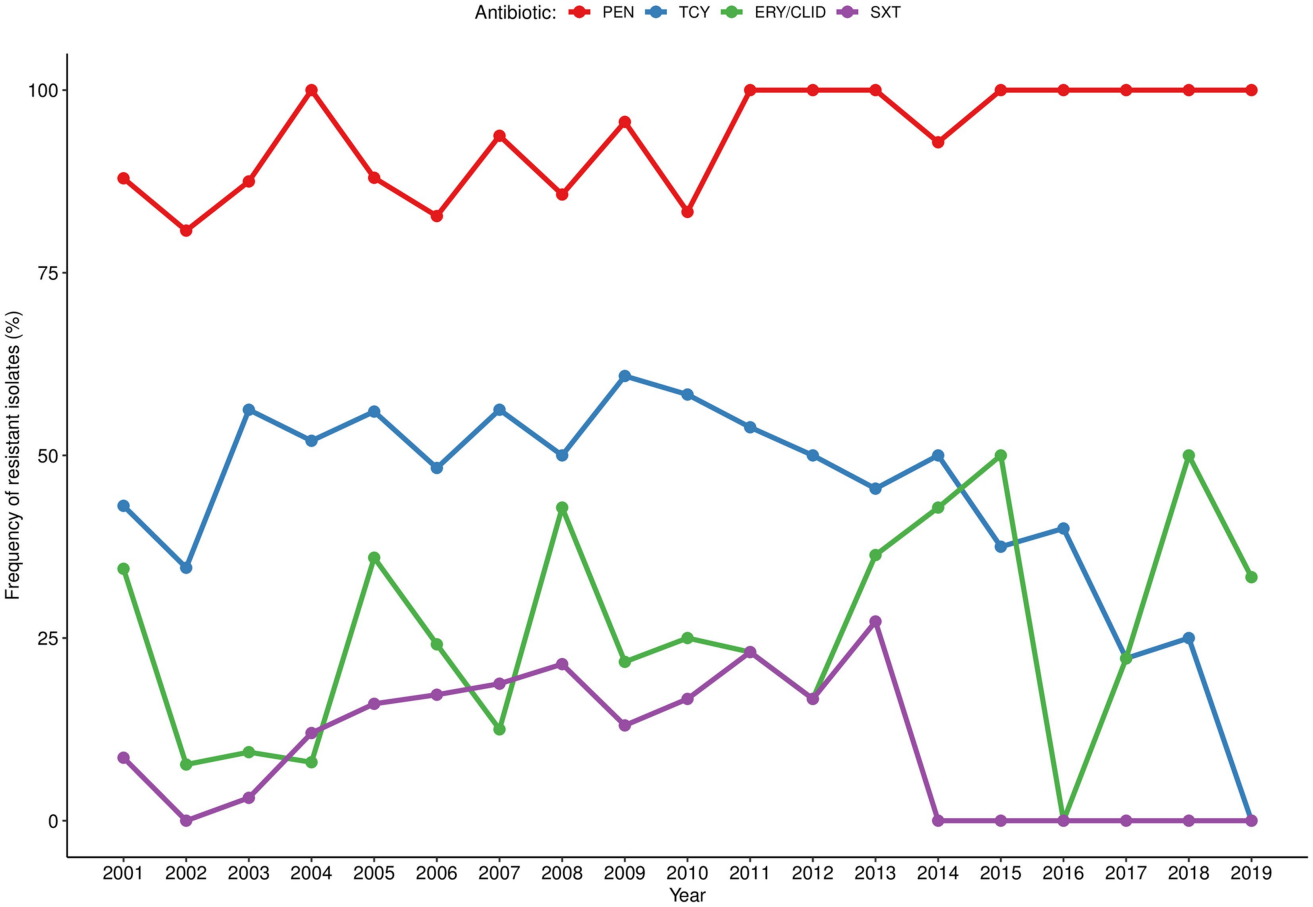
Temporal distribution of antibiotic resistance rates among *Staphylococcus aureus* isolated in children with bacteraemia. Resistant strains correspond to those presenting not susceptibility phenotype (resistant or intermediate). PEN, penicillin; TCY, tetracycline; ERY/CLID, erythromycin/clindamycin; SXT, co-trimoxazole.

**Figure 2:**
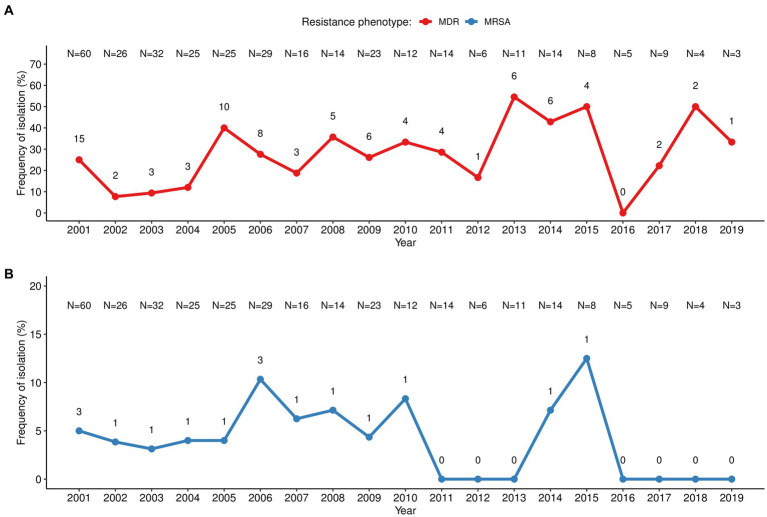
Temporal distribution of multiresistant *S. aureus* isolated in children with bacteraemia. **(A)** MDR, multidrug resistant *S. aureus* defined as those not susceptible to ≥3 unrelated classes of antibiotics; **(B)** MRSA, methicillin-resistant *S. aureus*. The number in each point (*n*) corresponds to the number of MDR/MRSA strains, while the number at the top (*N*) corresponds to the total number of *S. aureus* related to bacteraemia isolated in that year.

The antibiotic resistance pattern varied over the two decades of study according to the antibiotic tested (Figure 1). The pattern of resistance to penicillin was the most stable throughout the years with rates above 80% and peaking (100%) at the beginning (2004), middle (2011–2013) and at the end of the study period (2015–2019). The resistance rates to tetracycline ranged between 35 and 61% from 2001 to 2009 and steadily reduced (following the low *S. aureus* isolation frequency) in the subsequent years with sporadic peaks. In turn, resistance to erythromycin/clindamycin varied considerably (0%–50%) throughout the entire period of the surveillance, with peaks in 2015 and 2018; while resistance to co-trimoxazole increased between 2002 and 2013 (0%–27%) and was not detected from 2014 onwards. MDR strains accounted for 25% of the *S. aureus* isolated in the first year of surveillance (2001), reaching 40% in 2005. MDR frequency varied considerably over the study period (between 0% in 2016 and 50% in 2013, 2015, and 2018), albeit the absolute frequency of MDR strains diminished, following the lower isolation of *S. aureus* (Figure 2A). MRSA frequency ranged from less than 10% in the first decade of surveillance (2001–2010) to 0% in the second decade (2011–2019), except for 2014 and 2015, in which a single MRSA was detected each year (Figure 2B).

### 3.4. Resistance determinants

A good correlation was found between a not susceptibility phenotype and the resistance determinants screened, with the level of agreement by Cohen’s *κ* test revealing a “substantial perfect agreement” for tetracycline [*κ* = 0.7, 95% CI (0.6–0.8)] and “almost perfect agreement” for penicillin and cefoxitin [*κ* = 0.9, 95% CI (0.9–1.0) and 1.0, 95% CI (1.0–1.0), respectively] (Supplementary Table S4). For instance, total concordance was found between cefoxitin resistance phenotype and genotype, and 303 (>99%) out of 304 strains not susceptible to penicillin carried the *blaZ* gene encoding for a beta-lactamase (Table 2). For tetracycline, 157 out of 161 (98%) strains not susceptible to this antibiotic carried at least one *tet* determinant (*tet*(K), *tet*(L) or *tet*(M)), of which twenty-four carried two determinants (*tet*(L)*-tet*(M) (*n* = 12)*, tet*(K)*-tet*(L) (*n* = 10)*, tet*(K)*-tet*(M) (*n* = 2)) and five strains carried the three genes screened. Regarding co-trimoxazole, 31 out of 36 (86%) strains not susceptible carried *dfrA*(S1) and/or *dfrG* genes (including 2 strains carrying both genes). For macrolides, 69 out of 84 (80%) strains not susceptible carried *erm*(C) and/or *msr*(A) (including 5 strains carrying both genes), while the *erm*(A) gene was not detected. Two-thirds of the strains resistant to chloramphenicol carried the *cat*_pC221_ gene. The single strain resistant to ciprofloxacin had mutations in the QRDR of GrlA ([S80F] and [S144P]) and GyrA ([S84A]) targets (Table 2).

**Table 2:** Comparison between the susceptibility phenotype and carriage of resistance determinants among the *S. aureus* characterized.

Antibiotic name	Resistance determinants	Susceptibility category *n*/*N* (%)		
		Resistant	Intermediate	Susceptible
Penicillin	*blaZ*	303/304 (>99)	NA	1/32 (3)
Cefoxitin	*mecA*	16/16 (100)	NA	0
Tetracycline^a^	*tet*(K)	119/156 (76)	3/5 (60)	9/175 (5)
	*tet*(L)	29/156 (19)	0/5 (0)	ND
	*tet*(M)	39/156 (25)	1/5 (20)	ND
Co-trimoxazole^b^	*dfr*A(S1)	12/32 (38)	0/4 (0)	ND
	*dfr*G	18/32 (56)	3/4 (75)	ND
Erythromycin^c^	*erm*(C)	63/69 (91)	4/15 (27)	ND
	*erm*(A)	0/69 (0)	0/15 (0)	ND
	*msr*(A)	6/69 (9)	1/15 (7)	ND
Gentamicin	*aac*A*-aph*D	11/11 (100)	3/4 (75)	ND
Chloramphenicol	*cat*_pC221_	10/15 (67)	0/1 (0)	ND
Ciprofloxacin	GrlA ([S80F] + [S144P]) *+* GyrA [S84A]	1/1 (100)	0	ND

NA, not applicable; ND, not determined.

a Three out of 156 strains resistant to tetracycline, and 1/5 strains with intermediate resistance were simultaneously negative to *tet*(K), *tet*(M) and *tet*(L) genes.

b Four out of 32 strains resistant to co-trimoxazole, and 1/4 with intermediate resistance were simultaneously negative to the *dfrA*(S1) and *dfr*(G) genes.

c Four out of 69 strains resistant to erythromycin, and 11/15 strains with intermediate resistant were simultaneously negative to the *erm*(C), *erm*(A) and *msr*(A) genes.

### 3.5. Molecular typing

The *spa* typing revealed a high genetic diversity (SID = 0.97, CI 95% [0.96–0.97]) with 80 different *spa* types found among the entire collection (*n* = 336), including two novel types (t19593 and t19871). The high diversity of the study collection was further confirmed by PFGE analysis (performed for a subset of 160 isolates), revealing that 78% (124/160) of the isolates typed were clustered in 30 pulsotypes and in 109 subtypes, with each subtype containing one to three isolates, while the remaining 22% (36/160) isolates corresponded to single pulsotypes (data not shown). Frequent *spa* types were t084 (8%) and t002 (7%), followed by t355 (6%), t186 (6%), t645 (5%) and t174 (5%). The remaining *spa* types corresponded to <5% of the isolates (one to fifteen isolates) (Table 3). In two cases, the morphologically distinct *S. aureus* isolated from the same blood culture belonged to the same *spa* type (t888 and t934, respectively) while in the third case they belonged to distinct *spa* types (t008 and t174).

**Table 3:** Clonal relatedness among *S. aureus* (*n* = 336) analysed by *spa* typing and MLST.

CC^a^	ST^b^	*Spa* type	MRSA *n*/*N* (%)	MDR *n*/*N* (%)
CC5 (*N* = 58)	ST5	t002, t010, t071, t105, t306, t5259, t579, t8470	0	6/58 (10)
	ST6	t701, t934, t1476, t2360		
	ST650	t002, t062, t1470, t4535		
	ST6101	t045		
	ST6304	t002		
	ST7081	t304		
CC8 (*N* = 56)	ST8	t008, t334, t1476, t5472	15/56 (27)	22/56 (39)
	ST72	t148, t3092		
	ST612	t064		
	ST770	t9045		
	ST7201	t487		
CC15 (*N* = 37)	ST15	t084, t085, t346, t1492, t11928	0	2/37 (5)
	ST1160	t085, t9045		
	ST1906	t4340		
	ST6996	t3370		
	ST7202	t084		
	ST7351	t774		
CC1 (*N* = 36)	ST1	t127, t174, t254, t1931, t5471, t8538, t10719	0	9/36 (25)
	ST188	t2883		
	ST573	t1839		
	ST805	t1476		
	ST1292	t3086		
	ST2139	t174		
	ST7355	t14473		
CC121 (*N* = 34)	ST121	t272, t317, t645, t1114	0	9/34 (26)
	ST2430	t645, t19593, t2793		
	ST6098	t3772		
	ST6102	t14460		
	ST6995	t2793		
	ST7350	t2793		
CC152 (*N* = 33)	ST152	t355, t888, t1096, t1299, t1931, t5047	0	12/33 (36)
CC88 (*N* = 26)	ST88	t186, t690, t1951, t4125, t5351, t6449	1/26 (4)	6/26 (23)
	ST2141	t18888		
	ST7078	t786		
CC80 (*N* = 16)	ST80	t376, t934, t12119	0	1/16 (6)
	ST6994	t1198		
	ST7082	t16489		
CC25 (*N* = 20)	ST25	t078, t19871, t2554, t258, t3662, t3772, t9045	0	16/20 (80)
CC22 (*N* = 6)	ST22	t891	0	1/6 (17)
CC45 (*N* = 8)	ST508	t015	0	1/8 (13)
	ST6997	t445		
CC12 (*N* = 3)	ST12	t888	0	0
CC97 (*N* = 1)	ST97	t426	0	0
Singletons (*N* = 2)	ST3502	t2767	0	0
	ST7349	t10294		

a CC, clonal complex.

b ST, sequence type.

**Figure 3:**
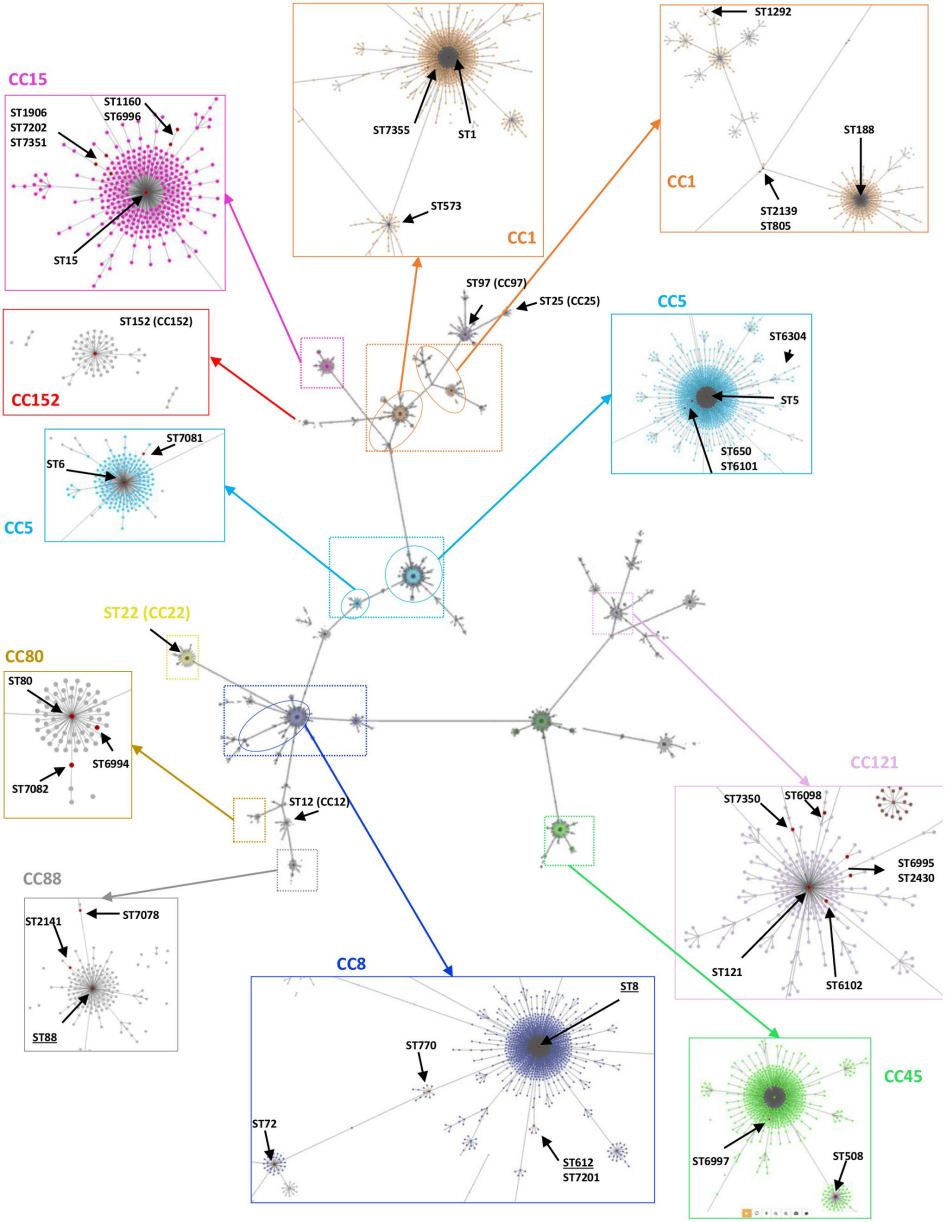
Overview of the clonal relatedness among *S. aureus* isolated in children with bacteraemia. The genetic relatedness was determined using PHYLOViZ Online software, including all the STs/CCs found in this current study plus the ones deposited in the PubMLST database until December 2022. Lines link all STs up to triple locus variants. The zoomed colored boxes highlight the clonal complexes (CCs) identified in this study, indicating all STs found by red dots. The underlined STs (e.g., ST8) indicate the ones including MRSA strains.

A subset of 168 *S. aureus*, representative of different *spa* types, was further analyzed by MLST, revealing 45 distinct STs (SID = 0.95, CI 95% [0.94-0.96]), including sixteen novel ones (ST6098, ST6101, ST6102, ST6304, ST6994, ST6995, ST6996, ST6997, ST7081, ST7082, ST7201, ST7202, ST7349, ST7350, ST7351, and ST7355). ST8 and ST25 predominated (12% and 10%, corresponding to 20 and 17 out of 168 isolates, respectively), followed by ST152 (7%), ST5 and ST612 (7% each), ST15 (6%), ST1 and ST88 (5% each). The remaining STs, including ST6, ST12, ST22, ST72, ST80, ST97, and ST121 corresponded to <5% of the collection (one to seven isolates) (Table 3). The novel STs corresponded to single (*n* = 12) or double (*n* = 3) locus variants of other STs circulating in Manhiça District. Forty-three STs clustered within thirteen CCs, while two STs (ST3502 and ST7349) were singletons, defined as those that did not match other STs at ≥4 loci (Figure 3). The clonal analysis of the entire *S. aureus* collection (determination of clonal complexes based on MLST for 168 isolates, and inference for the remaining 168 isolates as described in the Methodology), revealed a predominance of CC5 and CC8 (~17%, each), followed by CC15, CC1, CC121, CC152, CC88, CC25, and CC80, with frequency rates varying between 5% and 11% (Table 3). CC12, CC22, CC45, and CC97 were represented by <3% of the isolates (one to eight isolates) (Table 3).

### 3.6. SCC*mec* typing

SCC*mec* typing revealed that 12 out of the 16 MRSA (75%) carried a SCC*mec* type IV, which corresponded to SCC*mec* subtype IVa (ST88/CC88) and subtype IVd (ST612/CC8) for one and eleven strains, respectively. The remaining four MRSA (25%, ST8/CC8) carried a non-typable SCC*mec* (SCC*mec*NT).

### 3.7. Temporal distribution of *Staphylococcus aureus* clones

**Figure 4:**
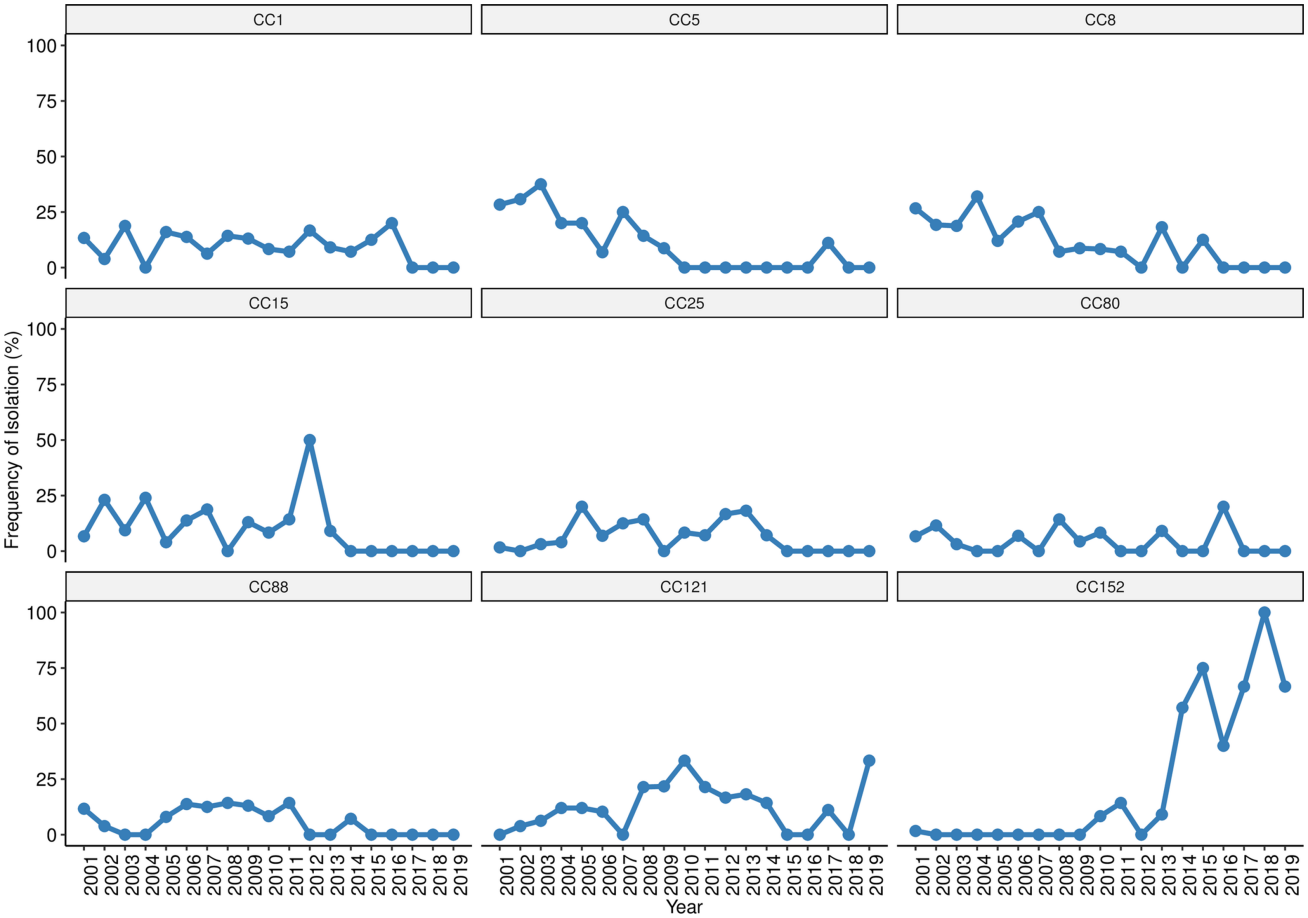
Trends of the most prevalent *S. aureus* clonal complexes isolated in children with bacteraemia.

Considering now the wider picture provided by the analysis of CCs, we observed an overall decrease of CC1, CC5, CC8, CC15, CC25, CC80, and CC88 throughout the surveillance and their absence in the last years (Figure 4). An opposing increasing trend was observed for CC121 and CC152, particularly for CC152 that was initially detected in 2001 and resurfaced in 2010 with remarkable increase throughout the remaining surveillance period, becoming the main clonal lineage of the last 6 years (Figure 4). Among the MRSA, clone t064-ST612/CC8-SCC*mec*IVd (69%, 11/16) was found only in the first nine years (2001, 2003–2009) of the surveillance, while clone t008-ST8/CC8 (25%, 4/16) harboring SCC*mec*NT was detected sporadically (2001, 2002, 2010, and 2015). A single MRSA strain from clone t5351-ST88/CC88-SCC*mec*IVa belonging to the “African clone” (Schaumburg et al., 2014) was isolated in 2014, thirteen years after the first detection of ST88-MSSA in our surveillance. The single ciprofloxacin resistant strain, which was detected in 2009, presented a MDR phenotype and belonged to clone t891-ST22/CC22. Strains from the same lineage (t891/CC22) but susceptible to ciprofloxacin and with a non-MDR/MSSA phenotype had been previously detected in 2006 (one strain), and then in 2009, 2011, 2016, and 2017 (one strain in each year).

### 3.8. Relatedness between *Staphylococcus aureus* clonal lineages and antibiotic resistance phenotypes

**Figure 5:**
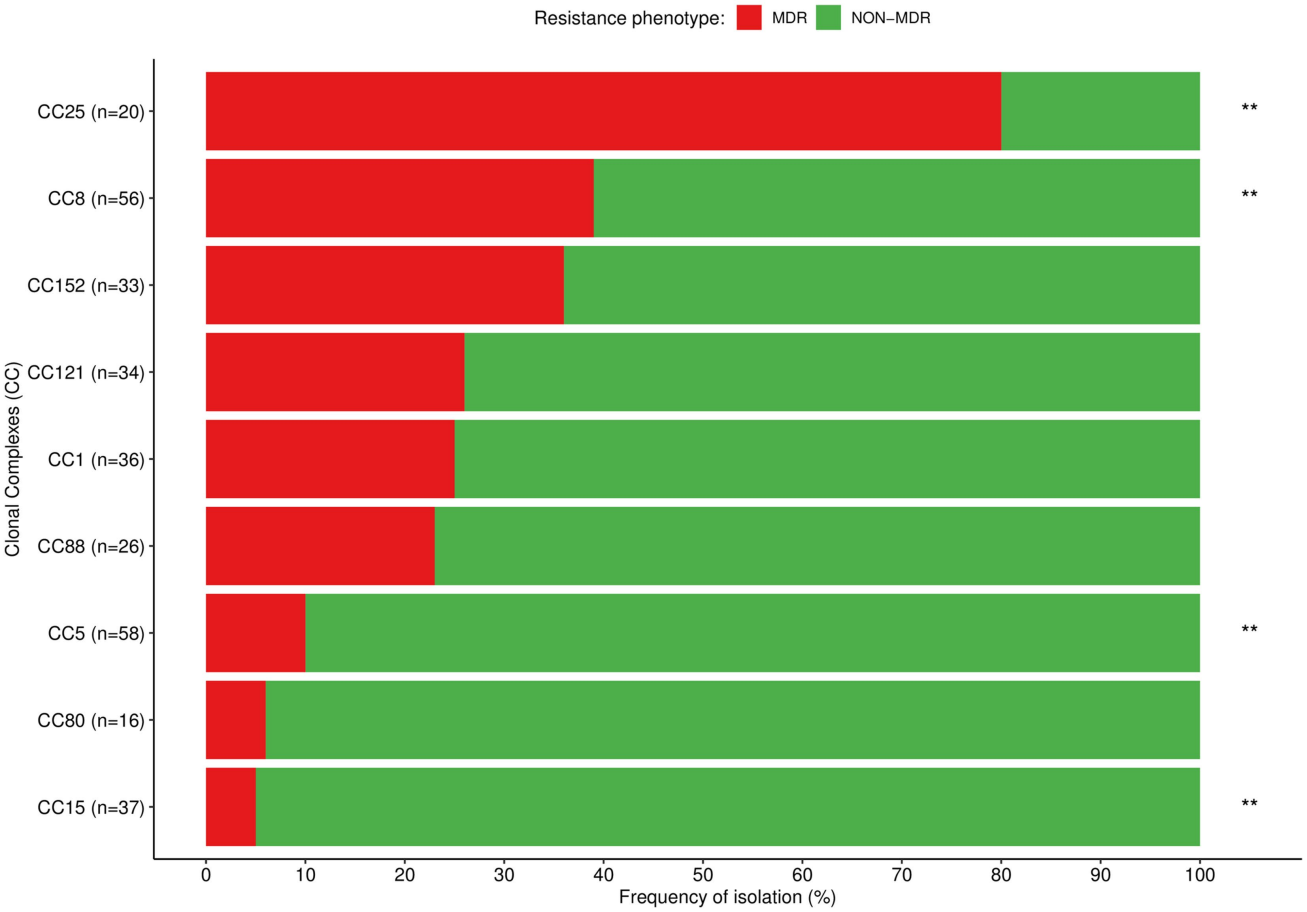
Distribution of the most prevalent clonal complexes among MDR (red) and non-MDR (green) *S. aureus* isolated in children with bacteraemia. Differences in the distribution of CCs between MDR and non-MDR strains were calculated with *χ*^2^ or Fisher’s exact test as appropriate; ^**^p < 0.01.

Our analysis revealed STs containing exclusively MRSA strains [ST612 (*n* = 11)], while others included both MRSA [ST8 (*n* = 4), ST88 (*n* = 1)] and MSSA strains [ST8 (*n* = 16), ST88 (*n* = 8)]. The MDR phenotype was predominantly observed among strains belonging to the ST612 [MDR (*n* = 11) *vs*. non-MDR (*n* = 0), *p* < 0.001] and ST25 [MDR (*n* = 13) *vs*. non-MDR (*n* = 4), *p* < 0.001], while non-MDR phenotype was predominantly or exclusively found among the remaining STs. Overlaying the CC data, MRSA belonged exclusively to CC8 and CC88, while MDR were commonly found among CC25. The MDR phenotype was also found in >35% of the strains from CC8 and CC152, and in >23% of CC1, CC88, and CC121. In contrast, non-MDR were significantly found among CC5, CC8, and CC15 (Table 3 and Figure 5).

Resistance to penicillin and penicillin-tetracycline were frequent among most CCs, while resistance to gentamicin was exclusively found among CC8 and CC22 (Table 4). Resistance to co-trimoxazole predominated among members from CC8 and CC25 and was less frequent among the CC1, CC5, CC22, CC88 and CC121. Similarly, resistance to chloramphenicol was highest among the CC8 and was less frequent among CC1 and CC25; while CC80 grouped most strains either fully susceptible or exclusively resistant to tetracycline (Table 4).

**Table 4:** Phenotypic resistance and resistance determinants among the most prevalent *S. aureus* clonal complexes.

CC	Resistance patterns (*n*)	Main resistance determinants (*n*)	MRSA *n* (%)	MDR *n* (%)
CC5 (*N* = 58)	PEN (31)	*blaZ* (31)	0	6 (10)
	PEN-TCY (14)	*blaZ*-*tet*(K) (10)		
	Others^a^ (8)	*blaZ-tet*(L)*-tet*(M)*-mrs*(A) (1)*, blaZ-tet*(K)*-erm*(C) (1)		
	Fully susceptible (5)	*blaZ* (1)		
CC8 (*N* = 56)	PEN (15)	*blaZ* (14)	15 (27)	22 (39)
	PEN-TCY (16)	*blaZ*-*tet*(K) (13)		
	FOX-PEN-TCY-GEN-SXT-CHL-ERY-CLID (9)	*blaZ-tet*(M)*-mecA-erm*(C)*-aacA_aphD-dfrA*(S1)*-cat* (5)		
	PEN-TCY-ERY-CLID (5)	*blaZ-tet*(L)*-tet*(M)*-erm*(C) (3)		
	Others^a^ (11)	*blaZ-tet*(*K*)*-dfrG* (2)		
CC15 (*N* = 37)	PEN (18)	*blaZ* (16)	0	2 (5)
	PEN-TCY (17)	*blaZ*-*tet*(K) (16)		
	Others^a^ (2)	*blaZ-tet*(K)*-erm*(C) (2)		
CC1 (*N* = 36)	PEN (14)	*blaZ* (12)	0	9 (25)
	PEN-TCY (7)	*blaZ*-*tet*(K) (5)		
	PEN-TCY-ERY-CLID (3)	*blaZ*-*tet*(K)-*erm*(C)-*msr*(A)		
	PEN-ERY-CLID (3)	*blaZ*-*erm*(C) (3)		
	TCY (2)	*tet*(*M*)		
	Others^a^ (7)	*blaZ* (*2*)		
	Fully susceptible (2)			
CC121 (*N* = 34)	PEN-TCY (11)	*blaZ-tet*(M) (7)	0	9 (26)
	PEN (10)	*blaZ* (10)		
	PEN-TCY-ERY-CLID (6)	*blaZ*-*tet*(M)-*erm*(C) (4), *blaZ*-*tet*(K)-*erm*(C) (2)		
	PEN-ERY (3)	*blaZ-erm*(C) (2)		
	Others^a^ (4)	*blaZ-erm*(C)*-dfrG, blaZ-tet*(*M*)*-dfrG*		
CC152 (*N* = 33)	PEN (14)	*blaZ* (14)	0	12 (36)
	PEN-ERY-CLID (8)	*blaZ* (*3*)*, blaZ-erm*(C) (3)*, blaZ-tet*(K)*-*		
	PEN-TCY (5)	*blaZ-tet*(K)*-tet*(L) (3)		
	PEN-TCY-ERY-CLID (4)	*blaZ-tet*(K)*-tet*(L)*-erm*(C) (1)*, blaZ-tet*(K)*-erm*(C) (1)		
	Fully susceptible (2)			
CC88 (*N* = 26)	PEN-TCY (14)	*blaZ-tet*(K) (13)	1 (4)	6 (23)
	PEN (2)	*blaZ* (2)		
	PEN-ERY (2)	*blaZ* (2)		
	PEN-TCY-ERY-CLID (2)	*blaZ-tet*(L)*-tet*(M)*-erm*(C) (*1*)*, blaZ-tet*(K) (1)		
	Others^a^ (6)	*blaZ-erm*(C) (2)		
CC25 (*N* = 20)	PEN-TCY-SXT-ERY-CLID (5)	*blaZ-tet*(K)*-erm*(C)*-dfrG* (5)	0	16 (80)
	PEN-SXT-ERY-CLID (3)	*blaZ-ermC-dfrG* (2)		
	PEN-TCY-SXT (3)	*blaZ-tet*(K)*-dfrG* (3)		
	PEN-SXT (2)	*blaZ-dfrG* (1)		
	Others^a^ (7)	*blaZ-tetK-erm*(C) (1)*, blaZ-erm*(C)*-cat* (1)		
CC80 (*N* = 16)	TCY (7)	*tet*(K) (7), *blaZ-tet*(K) (1)	0	1 (6)
	PEN-ERY-CLID (1)	*blaZ-erm*(C) (1)		
	PEN-TCY (1)	*blaZ-tet*(K) (1)		
	Fully susceptible (7)			

PEN, penicillin; FOX, cefoxitin; TCY, tetracycline; ERY, erythromycin; CLI, clindamycin; SXT, co-trimoxazole; CHL, chloramphenicol; GEN, gentamicin.

a Resistance profiles corresponding to ≤5% of isolates are detailed in Supplementary Table S5. PEN, penicillin; FOX, cefoxitin; TCY, tetracycline; ERY, erythromycin; CLI, clindamycin; SXT, co-trimoxazole; CHL, chloramphenicol; GEN, gentamicin.

### 3.9. Comparison between clonal lineage and clinical outcome

The comparative analysis of the microbiological data with available clinical records for length of stay, LOS (*n* = 279), revealed that children infected with strains from CC1, CC8, CC15, CC22, CC80, and CC152 had extended LOS (≥5 days) compared to those infected with strains from CC5, CC12, CC25, CC45, CC88, and CC121 (5 days, IQR, 3-8 *vs*. 4 days, IQR, 2-7, respectively, *p* = 0.0032). SAB caused by strains of CC8 was associated with mortality (18%, 9/49 *vs*. 7%, 16/230 for other CCs, *p =* 0.023), while no statistical difference was found for other CCs. Considering the available clinical records, CC8 (49 out of 56 CC8 strains with clinical data) was the only clonal lineage in which infection by MDR strains was associated to mortality compared to non-MDR (4%, 1/28 for non-MDR *vs*. 38% for MDR, 8/21, *p* = 0.003), but no significant difference was found between mortality and infection by MRSA within this specific clone (11%, 4/35 for MSSA *vs*. 36%, 5/14 for MRSA, *p* = 0.096). SAB by CC22 was exclusively found among infants and toddlers, while the CC1, CC5, CC8, CC45, and CC80 predominated among infants; and the CC25 and CC152 were found similarly distributed throughout all the age strata. All CCs but CC152 predominated in the rainy season (69%, 209/301 for other CCs *vs*. 47%, 15/32 for CC152, *p* = 0.010) (Table 5).

**Table 5:** *S. aureus* clonal complex and clinical outcome among children admitted with SAB, stratified by age and rainy season.

*S. aureus* clonal complex (CC)^a^	Length of hospital admission	Case fatality rate by CC^c^^,^^d^	Age category *n*/*N* (%)				Rainy season^c^
	Median days (IQR)^b^^,^^c^	*n*/*N*^d^ (%)	0–28 d	29 d–11 m	12–23 m	24–59 m	*n*/*N* (%)
CC8	7 (4–10)	9/49 (18)	15/56 (27)	20/56 (36)	11/56 (20)	10/56 (18)	38/56 (68)
CC80	6 (2–8)	0/15 (0)	6/16 (38)	7/16 (44)	2/16 (13)	1/16 (6)	10/16 (63)
CC1	5 (3–7)	4/30 (13)	11/35 (31)	11/35 (31)	8/35 (23)	5/35 (14)	24/35 (69)
CC15	5 (3–7)	3/35 (9)	14/37 (38)	7/37 (19)	13/37 (35)	3/37 (8)	28/37 (78)
CC22	5 (0–8)	0/2 (0)	0	4/6 (67)	2/6 (33)	0	4/6 (67)
CC152	5 (2–7)	1/23 (4)	9/32 (28)	7/32 (22)	10/32 (31)	6/32 (19)	15/32 (47)
CC121	4 (3–7)	3/29 (10)	12/34 (35)	5/34 (15)	7/34 (21)	10/34 (29)	21/34 (62)
CC25	4 (3–8)	1/16 (6)	4/20 (20)	7/20 (35)	5/20 (25)	4/20 (20)	13/20 (65)
CC5	4 (2–5)	3/50 (6)	21/57 (37)	14/57 (25)	11/57 (19)	11/57 (19)	40/57 (70)
CC88	3 (2–5)	0/20 (0)	7/26 (27)	6/26 (23)	9/26 (35)	4/26 (15)	21/26 (81)
CC45	3 (2–4)	1/6 (17)	3/8 (38)	2/8 (25)	2/8 (25)	1/8 (13)	4/8 (50)

a Data of groups with ≤3 isolates were not shown (CC12, CC97, and singletons).

b IQR: interquartile range.

c Data from Garrine et al. (2023).

d Complete records for mortality were available for 279 patients out of 333 children with SAB. d, days; m, months.

## 4. Discussion

### 4.1. High antibiotic resistance rates among bacteraemic *Staphylococcus aureus*

We performed a comprehensive characterization of the largest collection of SAB-related *S. aureus* strains documented so far in the Manhiça district, Mozambique. Our results showed a high genetic diversity of the *S. aureus* collection and a significant resistance burden, with circulation of 25% of MDR and a few MRSA strains that pose major challenges for the success of antimicrobial therapy in our setting, where the availability of second-line antibiotics is limited. Noteworthy, this study has reported the emergence and predominance of MDR and PVL-positive CC152 MSSA, a clonal lineage prevalent in the European continent (PVL-positive CA-MRSA), the Caribbean and the African continent (PVL-positive CA-MSSA) (Sowash and Uhlemann, 2014; Baig et al., 2020). Recent studies registered the circulation of PVL-positive CC152 MRSA in regions not previously detected (Democratic Republic of the Congo, Kenya, Nigeria and South Africa) (Lawal et al., 2022).

Data from local studies in Mozambique revealed distinct frequencies of circulating MDR and MRSA strains, with some studies from our setting (Sigaúque et al., 2009; Mandomando et al., 2010; Vubil et al., 2017) and other regions (Ceccarelli et al., 2005; van der Meeren et al., 2014) matching our data, while others reported significantly higher rates (Kenga et al., 2021). Despite the low rate of MRSA in our setting, our findings must be monitored with caution, as countries such as Tanzania, which initially reported a low prevalence of MRSA, saw a subsequent abrupt increase in their incidence (Mzee et al., 2021).

The high rate of penicillin resistance in our study is worrisome as this antibiotic (or ampicillin) in combination with gentamicin are empirically prescribed for hospital admitted patients with suspected invasive bacterial disease in Mozambique. The low resistance rate observed against gentamicin and chloramphenicol (the later less prescribed due to its toxicity, despite occasional use when other antibiotics stock out) suggest that these ready available antibiotics in our setting are still effective against *S. aureus.* Also, our data on MRSA and MDR frequencies supports ceftriaxone as a therapeutic alternative (Garrine et al., 2023). The high resistance rates observed to tetracycline was unexpected, considering its contraindication for administration in children <8 years, and probably reflects the misuse of this antibiotic outside the hospital environment. Previous studies from our setting reported significant proportion of informal antibiotic suppliers (non-licensed providers) (Do et al., 2021), common practice of self-medication and improper storage of medicines for unsupervised reuse (Cambaco et al., 2020). The low resistance rates for tetracycline and co-trimoxazole observed at the end of the surveillance period may reflect the overall reduction of SAB incidence observed by that time (Garrine et al., 2023) rather than changes on antimicrobial susceptibility patterns. Similar rates of resistance towards penicillin and tetracycline were previously reported for *S. aureus* of human or veterinary origin in Mozambique (Vubil et al., 2017; Nhatsave et al., 2021) and other African countries (Kolawole et al., 2013; Seni et al., 2013; Egyir et al., 2014; Eyasu et al., 2015; Dekker et al., 2016; Mekonnen et al., 2018). Contrarily, the resistance to co-trimoxazole in our study was lower (11%) comparing to previous reports for *S. aureus* in our setting (36%–69%) (Sigaúque et al., 2009; Mandomando et al., 2010; Vubil et al., 2017). This difference can reflect the lower number of isolates and shorter period of analysis in those previous reports. Nevertheless, the resistance trend of this antibiotic should be monitored, as co-trimoxazole prophylaxis is one of the key interventions among HIV-infected individuals in resource-limited settings (Saadani Hassani et al., 2015; Ministério da Saúde-Mozambique, 2016), including in Mozambique, where the prevalence of HIV/AIDS is among the highest in the world (Ministério da Saúde-Mozambique, 2021). In addition, sulfadoxine-pyrimethamine, an analogue drug of co-trimoxazole (antifolate drugs) has been extensively used for malaria prevention in HIV-negative pregnant women (WHO, 2021).

Most of the resistance determinants identified in our study are known to be carried in mobile genetic elements. This is an additional point of concern, taking into consideration that these may be transferred between different *S. aureus* strains or between *S. aureus* and other bacteria. Additionally, many of these mobile genetic elements (plasmids, transposons, SCC*mec*) may carry additional resistance determinants that can build up multiresistance patterns (Haaber et al., 2017; Partridge et al., 2018) and be easily transferred between strains.

### 4.2. Diversity of *Staphylococcus aureus* circulating in Manhiça District

Our analysis, covering *S. aureus* isolates recovered over two decades of surveillance (2001–2019), revealed circulation of distinct clones, as previously described regionally (Breurec et al., 2011; Schaumburg et al., 2014; Ruffing et al., 2017). The predominant CCs from our study (CC1, CC5, CC8, CC15, CC25, CC121, and CC152) correlated with the ones previously reported in multicenter studies involving several African countries (Breurec et al., 2011; Ruffing et al., 2017). The underlying reasons for the emergence of CC152 and declining of most of others CCs in the last years of the surveillance are still not understood; some studies suggest the competition between different clones and species as one of the factors that favor this expansion in a specific geographic area (Ruffing et al., 2017; Lakhundi and Zhang, 2018). Moreover, the evolution of CC152 mimics in many ways the genotypic and spatial characteristics of the European CC80 CA-MRSA clone, by its emergence from a PVL-positive MSSA ancestor from North Africa or Europe (Stegger et al., 2014; Baig et al., 2020). The PVL-positive CC152 CA-MRSA was rarely reported outside the European continent, while PVL-positive CC152 MSSA strains was associated with the African continent and the Caribbean, and less often in Europe (Baig et al., 2020). A recent report on the clonal distribution trend of MRSA across 16 African countries revealed overtime dissemination of CC1, CC22, and CC152 not previously found in specific locations (Lawal et al., 2022). Although all CC152 *S. aureus* from our study were MSSA, they should be monitored as a potential emerging CC.

Most MRSA strains in our study belonged to CC8, frequently associated with global outbreaks (Lee et al., 2018), with predominance of t064-ST612/CC8-SCC*mec*IVd and t008-ST8/CC8-SCC*mec*NT reflecting the clonal nature of the MRSA strains circulating in Manhiça. The ST612 is a double locus variant of the major clones USA500/CC8, a HA-MRSA strain (Carrel et al., 2015), and USA300/CC8, an epidemic CA-MRSA (Planet, 2017). The geographical distribution of ST612 is limited, being only described in specific regions of South Africa (Moodley et al., 2010; Jansen van Rensburg et al., 2011; Oosthuysen et al., 2014; Perovic et al., 2015, 2017; Lawal et al., 2022), Tanzania (Moremi et al., 2019) and Australia (Axon et al., 2011; Groves et al., 2016), frequently associated with veterinary practices (Groves et al., 2016; Murphy et al., 2018, 2019; Amoako et al., 2019). On the other hand, clone ST8-SCC*mec*IV has been frequently reported both in hospital and community settings in Angola, Cameroon, Gabon, Ghana, Madagascar, Nigeria, and São Tomé and Príncipe (Abdulgader et al., 2015). The ST88, also known as “African clone” is homogenously distributed across the continent being predominantly MRSA (Schaumburg et al., 2014; Ruffing et al., 2017; Lawal et al., 2022); however, in our study all but one (t5351-ST88-SCC*mec*IVa) of the strains belonging to the ST88 were MSSA. Recent report from our setting revealed circulation of human-adapted strains among *S. aureus* isolated from raw dairy milk samples, raising the hypothesis of potential anthroponotic transmission (Nhatsave et al., 2021). Further studies may include samples from different animal species and farmers in close contact to clarify the transmission dynamics of *S. aureus* between hosts. Despite the declining trend of CC5 (mostly represented by the ST5) in the last years of surveillance in our study, its circulation should be monitored as some studies reported the emergence of ST5-MRSA through the acquisition of the SCC*mec* element by the ST5-MSSA in Africa (Jansen van Rensburg et al., 2011; Schaumburg et al., 2014). This worrisome clone (ST5-MRSA), has been reported in South Africa (Moodley et al., 2010; Jansen van Rensburg et al., 2011), a border country of Mozambique. We identified novel STs that differed in one to two-point mutations from other STs circulating in Manhiça, suggesting that they evolved from the respective related ancestors. The limitation to type some *SCCmec* may originate on the protocol followed in our study that detects only eight (Zhang et al., 2005) out of fourteen SCC*mec* types and subtypes known to date (IWG-SCC, 2021), or result from the emergence of novel SCC*mec* structural variants.

Overall, the resistance rates were homogenously distributed among distinct CCs in our setting. Noteworthy, some exceptions were observed in which resistance to gentamicin, co-trimoxazole or chloramphenicol were related to specific *S. aureus* clonal complexes, calling for urgent monitoring of its trend. Of concern, these CCs are the ones that included MRSA strains (CC8), quinolone resistant strains (CC22) and significant number of MDR strains (CC25). We reported for the first time in Mozambique the circulation of *S. aureus* ST22 ciprofloxacin resistant carrying mutations in the QRDR of the target GrlA [S80F] and GyrA [S84A] subunits of the DNA Topoisomerase IV and DNA gyrase; in addition to a non-common mutation in GrlA [S144P], suspected to be a genetic polymorphism found both in susceptible and resistant strains (Cabrera et al., 2020). ST22 is one of the most common MRSA lineage healthcare-associated in Europe (Holden et al., 2013; Toleman et al., 2017; Lee et al., 2018), with subsequent spread into the community (Toleman et al., 2017). Therefore, there is a need to extend the ongoing morbidity surveillance to nosocomial infections for early detection and control of main strains of concern circulating in the hospital environment.

### 4.3. Impact of infection by specific CCs in patient outcome

The poor outcome among children infected by CC8 (a clone with global dissemination) in our study, suggests the potential of some clones to cause more severe disease (Recker et al., 2017; Horváth et al., 2020). Additionally, infection by CC8 MDR strains was associated to mortality, possibly in relation to resistance to the first line of empirical treatment (Garrine et al., 2023). This calls for a prompt recognition of SAB by specific clones to allow better clinical management of patients. Future studies should explore the virulence potential of these strains, and their interaction with human and animal hosts.

## 5. Conclusion

We found high diversity of bacteraemic *S. aureus* with high burden of MDR strains posing major challenges for the success of antimicrobial therapy in our setting. Specific clonal lineages were associated with poorer outcomes, in addition to the emergence of important *S. aureus* lineages.

## Data availability statement

The original contributions presented in the study are included in the article/Supplementary material, further inquiries can be directed to the corresponding author.

## Ethics statement

The studies involving human participants were reviewed and approved by Institutional Ethics Review Board for Health (CIBS), Centro de Investigação em Saúde de Manhiça (CISM), Maputo, Mozambique; National Bioethics Committee for Health (CNBS), Maputo, Mozambique. Written informed consent to participate in this study was provided by the participants’ legal guardian/next of kin.

## Author contributions

MG, SC, IC, and IM: conceptualization, data curation and writing-original draft. IM and IC: funding acquisition and resources. MG, SC, AM, SM, DV, SÁ, TN, IM, QB, and IC: investigation and methodology. MG, SC, IM, and IC: project administration. SC, IM, and IC: supervision. MG: formal analysis. MG and SC: software. MG, SC, AM, SM, DV, SÁ, TN, QB, IM, and IC: visualization, validation, and writing-review and editing. All authors contributed to the article and approved the submitted version.

## Funding

CISM receives core funding from “Agencia Española de Cooperacion Internacional para el Desarollo (AECID).” MG was supported by grant 145278, from Fundação Calouste Gulbenkian “Calouste Gulbenkian Foundation.” Additional support was provided by Fundação para a Ciência e a Tecnologia (FCT, Portugal) through funds to GHTM (UID/04413/2020). This study was partly supported by funds from PATH through to the pneumonia and pneumococcus surveillance study (GAT.770-790-01350-LPS), Bill & Melinda Gates Foundation through Center for Vaccine Development, University of Maryland School of Medicine, the United States Agency for International Development mission in Mozambique through to Fixed Obligation grant no. AID-656-F-12-00001, under RFA-656-12-000003, and the “Child Health and Mortality Prevention Surveillance-CHAMPS” through Bill & Melinda Gates Foundation under the grant OPP1126780, subcontract SC00003286. ISGlobal acknowledges support from the grant CEX2018-000806-S funded by MCIN/AEI/10.13039/501100011033, and support from the Generalitat de Catalunya through the CERCA Program.”

## Conflict of interest

The authors declare that the research was conducted in the absence of any commercial or financial relationships that could be construed as a potential conflict of interest.

## Publisher’s note

All claims expressed in this article are solely those of the authors and do not necessarily represent those of their affiliated organizations, or those of the publisher, the editors and the reviewers. Any product that may be evaluated in this article, or claim that may be made by its manufacturer, is not guaranteed or endorsed by the publisher.

## Footnotes

^1^ https://pubmlst.org/

^2^ https://www.graphpad.com/quickcalcs/kappa1/

^3^ http://darwin.phyloviz.net/

## Abbreviations

CISM: Centro de Investigação em Saúde de Manhiça
MDH: Manhiça District Hospital
