# Supplementary material for: Antimicrobial resistance and clonality of Staphylococcus aureus causing bacteraemia in children admitted to the Manhiça District Hospital, Mozambique, over two decades
Source: Front Microbiol. 2023 Jul 24;14:1208131. doi: 10.3389/fmicb.2023.1208131 (PMC10406509; doi:10.3389/fmicb.2023.1208131)
Supplement: Supplementary file 1 [file Table_1.docx]

***Supplementary Material***

**Title:** Antimicrobial resistance and clonality of *Staphylococcus aureus* causing bacteraemia in children admitted to the Manhiça District Hospital, Mozambique, over two decades

**Authors:** Marcelino Garrine^1,2^, Sofia Santos Costa^2^, Augusto Messa Jr^1^, Sérgio Massora^1^, Delfino Vubil^1^, Sozinho Ácacio^1,3^, Tacilta Nhampossa^1,3^, Quique Bassat^1,4,5,6,7^, Inácio Mandomando^1,3,4^ and Isabel Couto^2*^

***Correspondence:**Isabel Couto

Email: [icouto@ihmt.unl.pt](mailto:icouto@ihmt.unl.pt)

| **Target Gene** | **Primers** | **Nucleotide Sequence (5’-3’)** | **Amplicon Size (bp)** | **Reference** |
| --- | --- | --- | --- | --- |
|  |  | ***S. aureus* identification** |  |  |
| *nuc* | nuc_Fw | GCGATTGATGGTGATACGGTT | 270 | (Brakstad et al., 1992) |
|  | nuc_Rv | AGCCAAGCCTTGACGAACTAAAGC |  |  |
|  |  | **Screening of resistance determinants** |  |  |
| *mecA* | mecA_Fw | GGTCCCATTAACTCTGAAG | 1040 | (Petinaki et al., 2001) |
|  | mecA_Rv | AGTTCTGCAGTACCGGATTTGC |  |  |
| *blaZ* | blaZ_Fw | GATAAGAGATTTGCCTATGC | 533 | (Milheiriço et al., 2011) |
|  | blaZ_Rv | GCATATGTTATTGCTTGACC |  |  |
| *erm*(A) | erm(A)_Fw | AAGCGGTAAACCCCTCTGAG | 442 | (Jensen et al., 2002) |
|  | erm(A)_Rv | AAGCGGTAAACCCCTCTGAG |  |  |
| *erm*(C) | erm(C)_Fw | TCGTAACTGCCATTGAAATA | 348 | (Costa et al., 2016) |
|  | erm(C)_Rv | TCACTTTAGGTTTAGGATGAAA |  |  |
| *msr*(A) | msr(A)_Fw | GATTGTCCCAAGCCAGTAAA | 445 | (Ferreira et al., 2021) |
|  | msr(A)_Rv | GCCATTTGCACTTTAGGAGA |  |  |
| *tet*(K) | tet(K)_Fw | GTAGCGACAATAGGTAATAGT | 361 | (Strommenger et al., 2003) |
|  | tet(K)_Rv | GTAGTGACAATAAACCTCCTA |  |  |
| *tet*(M) | tet(M)_Fw | GTTAAATAGTGTTCTTGGAG | 657 | (Aarestrup et al., 2000) |
|  | tet(M)_Rv | CTAAGATATGGCTCTAACAA |  |  |
| *tet*(L) | tet(L)_Fw | GTCGGTAATTGGGTTTGTTG | 421 | (Costa et al., 2021) |
|  | tet(L)_Rv | TGACAGCACGCTAACGATAA |  |  |
| *aacA-aphD* | aacA-aphD_Fw | CAGAGCCTTGGGAAGATGAAG | 348 | (Vakulenko et al., 2003) |
|  | aacA-aphD_Rv | CCTCGTGTAATTCATGTTCTGGC |  |  |
| *dfrG* | dfrG_Fw | TTTCTTTGATTGCTGCGATG | 501 | (Couto et al., 2014) |
|  | dfrG_Rv | AACGCACCCGTTAACTCAAT |  |  |
| *dfrA*(S1) | dfrA(S1)_Fw | CACTTGTAATGGCACGGAAA | 270 | (Argudín et al., 2011) |
|  | dfrA(S1)_Rv | CGAATGTGTATGGTGGAAAG |  |  |

**Table S1.** **Primers used in this study.**

**Table S1. *(Cont.)*** **Primers used in this study.**

| **Target Gene** | **Primers** | **Nucleotide Sequence (5’-3’)** | **Amplicon Size (bp)** | **Reference** |
| --- | --- | --- | --- | --- |
| *grlA* | grlA_Fw | CAAGAGCGTGCTTTRCCT | 300 | (Costa et al., 2021) |
|  | grlA_Rv | CTGACTYAATTTCGCTTCAG |  |  |
| *gyrA* | gyrA_Fw | ATGAGTGTTATYGTRTCTCGT | 261 |  |
|  | gyrA_Rv | CATMGAACCRAAGTTACCTTG |  |  |
| *cat*p*_C221_* | catpC221_Fw | ATTTATGCAATTATGGAAGTTG | 435 | (Schnellmann et al., 2006) |
|  | catpC221_Rv | TGAAGCATGGTAACCATCAC |  |  |
|  |  | ***spa* typing** |  |  |
| *spa* | spa-1113-Fw | TAAAGACGATCCTTCGGTGAGC | 110-442 | (Aires-de-Sousa et al., 2006) |
|  | spa-1514-Rv | CAGCAGTAGTGCCGTTTGCTT |  |  |
|  |  | **MLST** |  |  |
| *arcC* | arcC_Fw | CCT TTATTTGATTCACCAGCG | 456 | (Crisostomo et al., 2001) |
|  | arcC_Rv | AGGTATCTGCTTCAATCAGCG |  | (Enright et al., 2000) |
| *aroE* | aroE_Fw | ATCGGAAATCCTATTTCACATTC | 456 | (Enright et al., 2000) |
|  | aroE_Rv | GGTGTTGTATTAATAACGATATC |  |  |
| *glpF* | glpF_Fw | CTAGGAACTGCAATCTTAATCC | 465 | (Enright et al., 2000) |
|  | glpF_Rv | TGGTAAAATCGCATGTCCAATTC |  |  |
| *gmk* | gmk_Fw | ATCGTTTTATCGGGACCATC | 429 | (Enright et al., 2000) |
|  | gmk_Rv | TCATTAACTACAACGTAATCGTA |  |  |
| *pta* | pta_Fw | GTTAAAATCGTATTACCTGAAGG | 474 | (Enright et al., 2000) |
|  | pta_Rv | GACCCTTTTGTTGAAAAGCTTAA |  |  |
| *tpi* | tpi_Fw | TCGTTCATTCTGAACGTCGTGAA | 402 | (Enright et al., 2000) |
|  | tpi_Rv | TTTGCACCTTCTAACAATTGTAC |  |  |
| *yqiL* | yqiL_Fw | CAGCATACAGGACACCTATTGGC | 516 | (Enright et al., 2000) |
|  | yqiL_Rv | CGTTGAGGAATCGATACTGGAAC |  |  |

|  |  | **SCC*mec* typing** |  |  |
| --- | --- | --- | --- | --- |
| SCC*mec*I | SCCmecI_Fw | GCTTTAAAGAGTGTCGTTACAGG | 613 | (Zhang et al., 2005) |
|  | SCCmecI_Rv | GTTCTCTCATAGTATGACGTCC |  |  |
| SCC*mec*II | SCCmecII_Fw | CGTTGAAGATGATGAAGCG | 398 | (Zhang et al., 2005) |
|  | SCCmecII_Rv | CGAAATCAATGGTTAATGGACC |  |  |
| SCC*mec*III | SCCmecIII_Fw | CCATATTGTGTACGATGCG | 280 | (Zhang et al., 2005) |
|  | SCCmecIII_Rv | CCTTAGTTGTCGTAACAGATCG |  |  |
| SCC*mec*IVa | SCCmecIVa_Fw | GCCTTATTCGAAGAAACCG | 776 | (Zhang et al., 2005) |
|  | SCCmecIVa_Rv | CTACTCTTCTGAAAAGCGTCG |  |  |
| SCC*mec*IVb | SCCmecIVb_Fw | TCTGGAATTACTTCAGCTGC | 493 | (Zhang et al., 2005) |
|  | SCCmecIVb_Rv | AAACAATATTGCTCTCCCTC |  |  |
| SCC*mec*IVc | SCCmecIVc_Fw | ACAATATTTGTATTATCGGAGAGC | 200 | (Zhang et al., 2005) |
|  | SCCmecIVc_Rv | TTGGTATGAGGTATTGCTGG |  |  |
| SCC*mec*IVd | SCCmecIVd_Fw | CTCAAAATACGGACCCCAATACA | 881 | (Zhang et al., 2005) |
|  | SCCmecIVd_Rv | TGCTCCAGTAATTGCTAAAG |  |  |
| SCC*mec*V | SCCmecV_Fw | GAACATTGTTACTTAAATGAGCG | 325 | (Zhang et al., 2005) |
|  | SCCmecV_Rv | TGAAAGTTGTACCCTTGACACC |  |  |

**bp:** base pair; **Fw:** “forward”; **Rv: “**reverse”; R: A + G; Y: C + T; M: A + C.

**References**

Aarestrup, F. M., Agerso, Y., Gerner–Smidt, P., Madsen, M., and Jensen, L. B. (2000). Comparison of antimicrobial resistance phenotypes and resistance genes in *Enterococcus faecalis* and *Enterococcus faecium* from humans in the community, broilers, and pigs in Denmark. *Diagn. Microbiol. Infect. Dis.* 37, 127–137. doi: 10.1016/S0732-8893(00)00130-9.

Aires-de-Sousa, M., Boye, K., de Lencastre, H., Deplano, A., Enright, M. C., Etienne, J., et al. (2006). High interlaboratory reproducibility of DNA sequence-based typing of bacteria in a multicenter study. *J. Clin. Microbiol.* 44, 619–21.

Argudín, M. A., Tenhagen, B.-A., Fetsch, A., Sachsenröder, J., Käsbohrer, A., Schroeter, A., et al. (2011). Virulence and Resistance Determinants of German *Staphylococcus aureus* ST398 Isolates from Nonhuman Sources. *Appl. Environ. Microbiol.* 77, 3052–3060. doi: 10.1128/AEM.02260-10.

Brakstad, O. G., Aasbakk, K., and Maeland, J. A. (1992). Detection of *Staphylococcus aureus* by polymerase chain reaction amplification of the nuc gene. *J. Clin. Microbiol.* 30, 1654–1660.

Costa, S. S., Oliveira, V., Serrano, M., Pomba, C., and Couto, I. (2021). Phenotypic and Molecular Traits of *Staphylococcus coagulans* Associated with Canine Skin Infections in Portugal. *Antibiotics* 10, 518. doi: 10.3390/antibiotics10050518.

Costa, S. S., Palma, C., Kadlec, K., Fessler, A. T., Viveiros, M., Melo-Cristino, J., et al. (2016). Plasmid-Borne Antimicrobial Resistance of *Staphylococcus aureus* Isolated in a Hospital in Lisbon, Portugal. *Microb. Drug Resist.* 22, 617–626. doi: 10.1089/mdr.2015.0352.

Couto, N., Belas, A., Couto, I., Perreten, V., and Pomba, C. (2014). Genetic Relatedness, Antimicrobial and Biocide Susceptibility Comparative Analysis of Methicillin-Resistant and - Susceptible *Staphylococcus pseudintermedius* from Portugal. *Microb. Drug Resist.* 20, 364–371. doi: 10.1089/mdr.2013.0043.

Crisostomo, M. I., Westh, H., Tomasz, A., Chung, M., Oliveira, D. C., and de Lencastre, H. (2001). The evolution of methicillin resistance in *Staphylococcus aureus*: Similarity of genetic backgrounds in historically early methicillin-susceptible and - resistant isolates and contemporary epidemic clones. *Proc. Natl. Acad. Sci.* 98, 9865–9870. doi: 10.1073/pnas.161272898.

Enright, M. C., Day, N. P., Davies, C. E., Peacock, S. J., and Spratt, B. G. (2000). Multilocus sequence typing for characterization of methicillin-resistant and methicillin-susceptible clones of *Staphylococcus aureus*. *J. Clin. Microbiol.* 38, 1008–15.

Ferreira, C., Costa, S. S., Serrano, M., Oliveira, K., Trigueiro, G., Pomba, C., et al. (2021). Clonal Lineages, Antimicrobial Resistance, and PVL Carriage of *Staphylococcus aureus* Associated to Skin and Soft-Tissue Infections from Ambulatory Patients in Portugal. *Antibiotics* 10, 345. doi: 10.3390/antibiotics10040345.

Jensen, L. B., Hammerum, A. M., Bager, F., and Aarestrup, F. M. (2002). Streptogramin Resistance among *Enterococcus faecium* Isolated from Production Animals in Denmark in 1997. *Microb. Drug Resist.* 8, 369–374. doi: 10.1089/10766290260469642.

Milheiriço, C., Portelinha, A., Krippahl, L., de Lencastre, H., and Oliveira, D. C. (2011). Evidence for a purifying selection acting on the β-lactamase locus in epidemic clones of methicillin-resistant *Staphylococcus aureus*. *BMC Microbiol.* 11, 76. doi: 10.1186/1471-2180-11-76.

Petinaki, E., Arvaniti, A., Dimitracopoulos, G., and Spiliopoulou, I. (2001). Detection of *mecA*, *mecR1* and *mecI* genes among clinical isolates of methicillin-resistant staphylococci by combined polymerase chain reactions. *J. Antimicrob. Chemother.* 47, 297–304. doi: 10.1093/jac/47.3.297.

Schnellmann, C.; Gerber, V.; Rossano, A.; Jaquier, V.; Panchaud, Y.; Doherr, M.G.; et al. (2006). Presence of new *mecA* and *mph*(C) variants conferring antibiotic resistance in *Staphylococcus* spp. isolated from the skin of horses before and after clinic admission. *J. Clin. Microbiol.* 44, 4444-4454. doi: 10.1128/JCM.00868-06

Strommenger, B., Kettlitz, C., Werner, G., and Witte, W. (2003). Multiplex PCR Assay for Simultaneous Detection of Nine Clinically Relevant Antibiotic Resistance Genes in *Staphylococcus aureus*. *J. Clin. Microbiol.* 41, 4089–4094. doi: 10.1128/JCM.41.9.4089-4094.2003.

Vakulenko, S. B., Donabedian, S. M., Voskresenskiy, A. M., Zervos, M. J., Lerner, S. A., and Chow, J. W. (2003). Multiplex PCR for Detection of Aminoglycoside Resistance Genes in Enterococci. *Antimicrob. Agents Chemother.* 47, 1423–1426. doi: 10.1128/AAC.47.4.1423-1426.2003.

Zhang, K., McClure, J.-A., Elsayed, S., Louie, T., and Conly, J. M. (2005). Novel Multiplex PCR Assay for Characterization and Concomitant Subtyping of *Staphylococcal* Cassette Chromosome *mec* Types I to V in Methicillin-Resistant *Staphylococcus aureus*. *J. Clin. Microbiol.* 43, 5026–5033. doi: 10.1128/JCM.43.10.5026-5033.2005.
