# Supplementary material for: Antimicrobial resistance and clonality of Staphylococcus aureus causing bacteraemia in children admitted to the Manhiça District Hospital, Mozambique, over two decades
Source: Front Microbiol. 2023 Jul 24;14:1208131. doi: 10.3389/fmicb.2023.1208131 (PMC10406509; doi:10.3389/fmicb.2023.1208131)
Supplement: Supplementary file 2 [file Table_2.docx]

Supplementary Material

**Title:** Antimicrobial resistance and clonality of *Staphylococcus aureus* causing bacteraemia in children admitted to the Manhiça District Hospital, Mozambique, over two decades

**Authors:** Marcelino Garrine^1,2^, Sofia Santos Costa^2^, Augusto Messa Jr^1^, Sérgio Massora^1^, Delfino Vubil^1^, Sozinho Ácacio^1,3^, Tacilta Nhampossa^1,3^, Quique Bassat^1,4,5,6,7^, Inácio Mandomando^1,3,4^ and Isabel Couto^2*^

***Correspondence:**Isabel Couto

Email: [icouto@ihmt.unl.pt](mailto:icouto@ihmt.unl.pt)

**Table S2. Results from previous studies showing similarity or agreement between results from *spa* typing and**

**MLST of *S. aureus.***

| **#** | ***spa* type** | **Sequence type** | **Clonal complex** | **Reference** |
| --- | --- | --- | --- | --- |
| 1 | t008 | ST8 | CC8 | (Silva et al., 2022) |
|  |  |  |  | (Goudarzi et al., 2020) |
|  |  |  |  | (Wang et al., 2022) |
|  |  | ST8 (+++)/ST247(+) |  | (Asadollahi et al., 2018) |
|  |  | ST8/ST113 |  | (Boswihi et al., 2016) |
|  |  | ST8 |  | (Goudarzi et al., 2021) |

**Table S2. *(Cont.)* Results from previous studies showing similarity or agreement between results from *spa* typing**

**and MLST of *S. aureus.***

| **#** | ***spa* type** | **Sequence type** | **Clonal complex** | **Reference** |
| --- | --- | --- | --- | --- |
| 2 | t064 | ST8 | CC8 | (Khan et al., 2021) |
|  |  |  |  | (Goudarzi et al., 2019) |
|  |  |  |  | (Boswihi et al., 2016) |
|  |  |  |  | (Goudarzi et al., 2020) |
| 3 | t084 | ST15 (+++) | CC15 | (Creutz et al., 2022) |
|  |  |  |  | (Asadollahi et al., 2018) |
|  |  |  |  | (Goudarzi et al., 2019) |
|  |  |  |  | (Sahin-Tóth et al., 2021) |
|  |  | ST1535 (+) |  | (Udo et al., 2020) |
| 4 | t002 | ST5 | CC5 | (Goudarzi et al., 2019) |
|  |  |  |  | (Boswihi et al., 2016) |
|  |  |  |  | (Asadollahi et al., 2018) |
| 5 | t701 | ST6 | CC5 | (He et al., 2021) |
|  |  |  |  | (Liao et al., 2020) |
| 6 | t127 | ST1 | CC1 | (Huang et al., 2021) |
|  |  |  |  | (He et al., 2021) |
|  |  |  |  | (Hait et al., 2021) |
|  |  |  |  | (Boudet et al., 2021) |
|  |  |  |  | (Boswihi et al., 2016) |
|  |  |  |  | (Petersen et al., 2021) |

**Table S2. *(Cont.)* Results from previous studies showing similarity or agreement between results from *spa* typing**

**and MLST of *S. aureus.***

| **#** | ***spa* type** | **Sequence type** | **Clonal complex** | **Reference** |
| --- | --- | --- | --- | --- |
| 7 | t1299 | ST152 | CC152 | (Nhatsave et al., 2021) |
| 8 | t148 | ST72 | CC8 | (Tavares et al., 2014) |
|  |  |  |  | (Garcia et al., 2016) |
| 9 | t186 | ST78 | CC88 | (Earls et al., 2018) |
|  |  | ST88 |  | (Ohadian Moghadam et al., 2017) |
|  |  |  |  | (Kpeli et al., 2016) |
| 10 | t355 | ST152 | CC152 | (Kpeli et al., 2016) |
|  |  |  |  | (Egyir et al., 2021) |
|  |  |  |  | (Egyir et al., 2020) |
|  |  |  |  | (Nyasinga et al., 2019) |
| 11 | t645 | ST121 | CC121 | (Haque et al., 2019) |
| 12 | t010 | ST149 | CC5 | (Mairi et al., 2021) |
|  |  | ST5 |  | (Rijnders et al., 2009) |
|  |  | ST2633 |  | (Li et al., 2019) |
| 13 | t174 | ST1 | CC1 | (Parisi et al., 2016) |
|  |  |  |  | (Normanno et al., 2015) |
| 14 | t376 | ST80 | CC80 | (Mairi et al., 2020) |
|  |  |  |  | (Boswihi et al., 2016) |
| 15 | t015 | ST45 | CC45 | (Kwapisz et al., 2020) |
|  |  | not identified (microarray typing) |  | (Morach et al., 2019) |
|  |  | ST45 |  | (Loncaric et al., 2019) |
|  |  | not identified (microarray typing) |  | (Deasy et al., 2019) |

**Table S2. *(Cont.)* Results from previous studies showing similarity or agreement between results from *spa* typing**

**and MLST of *S. aureus.***

| **#** | ***spa* type** | **Sequence type** | **Clonal complex** | **Reference** |
| --- | --- | --- | --- | --- |
| 16 | t1198 | ST80 (SLV of ST6994) | CC80 | (Islam et al., 2019) |
|  |  |  |  | (Aung et al., 2017) |
| 17 | t2793 | not identified (microarray typing) | CC45 | (Vubil et al., 2017) |
|  |  |  | CC121 |  |
| 18 | t317 | ST121 | CC121 | (Breurec et al., 2011) |
| 19 | t888 | ST12 |  | (Nethercott et al., 2013) |
|  |  | not determined |  | (Garbacz et al., 2021) |
| 20 | t891 | ST22 | CC22 | (Antiabong et al., 2017) |
| 21 | t1476 | ST8 | CC8 | (Lebughe et al., 2017) |
|  |  |  |  | (Schaumburg et al., 2015) |
|  |  |  |  | (Vandendriessche et al., 2017) |
| 22 | t3772 | ST25 | CC25 | (Schaumburg et al., 2011) |
|  |  | not identified (microarray typing) | CC25 | (Vubil et al., 2017) |

**(+++)**: Most common sequence type (ST) identified among specific *spa* type; **(+)** less predominant ST among specific *spa* type.

**References**

Antiabong, J. F., Kock, M. M., Maphanga, T. G., Salawu, A. M., Mbelle, N. M., and Ehlers, M. M. (2017). Trends in the Genetic Background of Methicillin-Resistant *Staphylococcus aureus* Clinical Isolates in a South African Hospital: An Institutional-Based Observational Study. *Open Microbiol. J.* 11, 339–351. doi: 10.2174/1874285801711010339.

Asadollahi, P., Farahani, N. N., Mirzaii, M., Khoramrooz, S. S., van Belkum, A., Asadollahi, K., et al. (2018). Distribution of the Most Prevalent *spa* types among Clinical Isolates of Methicillin-Resistant and-Susceptible *Staphylococcus aureus* around the World: A Review. *Front. Microbiol.* 9, 163. doi: 10.3389/fmicb.2018.00163.

Aung, K. T., Hsu, L. Y., Koh, T. H., Hapuarachchi, H. C., Chau, M. L., Gutiérrez, R. A., et al. (2017). Prevalence of methicillin-resistant *Staphylococcus aureus* (MRSA) in retail food in Singapore. *Antimicrob. Resist. Infect. Control* 6, 94. doi: 10.1186/s13756-017-0255-3.

Boswihi, S. S., Udo, E. E., and Al-Sweih, N. (2016). Shifts in the Clonal Distribution of Methicillin-Resistant *Staphylococcus aureus* in Kuwait Hospitals: 1992-2010. *PLOS ONE* 11, e0162744. doi: 10.1371/journal.pone.0162744.

Boudet, A., Jay, A., Dunyach-Remy, C., Chiron, R., Lavigne, J.-P., and Marchandin, H. (2021). In-Host Emergence of Linezolid Resistance in a Complex Pattern of Toxic Shock Syndrome Toxin-1-Positive Methicillin-Resistant *Staphylococcus aureus* Colonization in Siblings with Cystic Fibrosis. *Toxins* 13, 317. doi: 10.3390/toxins13050317.

Breurec, S., Fall, C., Pouillot, R., Boisier, P., Brisse, S., Diene-Sarr, F., et al. (2011). Epidemiology of methicillin-susceptible *Staphylococcus aureus* lineages in five major African towns: high prevalence of Panton-Valentine leukocidin genes. *Clin. Microbiol. Infect.* 17, 633–639. doi: 10.1111/j.1469-0691.2010.03320.x.

Creutz, I., Busche, T., Layer, F., Bednarz, H., Kalinowski, J., and Niehaus, K. (2022). Evaluation of virulence potential of methicillin-sensitive and methicillin-resistant *Staphylococcus aureus* isolates from a German refugee cohort. *Travel Med. Infect. Dis.* 45, 102204. doi: 10.1016/j.tmaid.2021.102204.

Deasy, E. C., Brennan, G. I., Tecklenborg, S. C., Umeh, C., Coleman, D. C., and Shore, A. C. (2019). A molecular epidemiological investigation of methicillin-susceptible *Staphylococcus aureus* causing bloodstream infections in Ireland, 2006–2017. *Eur. J. Clin. Microbiol. Infect. Dis.* 38, 927–936. doi: 10.1007/s10096-019-03523-0.

Earls, M. R., Coleman, D. C., Brennan, G. I., Fleming, T., Monecke, S., Slickers, P., et al. (2018). Intra-Hospital, Inter-Hospital and Intercontinental Spread of ST78 MRSA From Two Neonatal Intensive Care Unit Outbreaks Established Using Whole-Genome Sequencing. *Front. Microbiol.* 9, 1485. doi: 10.3389/fmicb.2018.01485.

Egyir, B., Bentum, J., Attram, N., Fox, A., Obeng-Nkrumah, N., Appiah-Korang, L., et al. (2021). Whole Genome Sequencing and Antimicrobial Resistance of *Staphylococcus aureus* from Surgical Site Infections in Ghana. *Pathogens* 10, 196. doi: 10.3390/pathogens10020196.

Egyir, B., Hadjirin, N. F., Gupta, S., Owusu, F., Agbodzi, B., Adogla-Bessa, T., et al. (2020). Whole-genome sequence profiling of antibiotic-resistant *Staphylococcus aureus* isolates from livestock and farm attendants in Ghana. *J. Glob. Antimicrob. Resist.* 22, 527–532. doi: 10.1016/j.jgar.2020.03.029.

Garbacz, K., Wierzbowska, M., Kwapisz, E., Kosecka-Strojek, M., Bronk, M., Saki, M., et al. (2021). Distribution and antibiotic-resistance of different *Staphylococcus* species identified by matrix assisted laser desorption ionization-time of flight mass spectrometry (MALDI-TOF MS) isolated from the oral cavity. *J. Oral Microbiol.* 13, 1983322. doi: 10.1080/20002297.2021.1983322.

Garcia, C., Acuña-Villaorduña, A., Dulanto, A., Vandendriessche, S., Hallin, M., Jacobs, J., et al. (2016). Dynamics of nasal carriage of methicillin-resistant *Staphylococcus aureus* among healthcare workers in a tertiary-care hospital in Peru. *Eur. J. Clin. Microbiol. Infect. Dis.* 35, 89–93. doi: 10.1007/s10096-015-2512-9.

Goudarzi, M., Fazeli, M., Eslami, G., and Pouriran, R. (2019). Genetic Diversity Analysis of Mupirocin-Resistant *Staphylococcus aureus* Clinical Isolates in Tehran Hospitals, Iran. *Microb. Drug Resist.* 25, 558–566. doi: 10.1089/mdr.2018.0146.

Goudarzi, M., Hajikhani, B., Nasiri, M. J., Goudarzi, H., Dadashi, M., Haghighi, M., et al. (2021). Emergence of CC8/ST239- SCCmec III/t421 tigecycline resistant and CC/ST22-SCCmec IV/t790 vancomycin resistant *Staphylococcus aureus* strains isolated from wound: A two-year multi-center study in Tehran, Iran. *Acta Microbiol. Immunol. Hung.* doi: 10.1556/030.2021.01534.

Goudarzi, M., Kobayashi, N., Dadashi, M., Pantůček, R., Nasiri, M. J., Fazeli, M., et al. (2020). Prevalence, Genetic Diversity, and Temporary Shifts of Inducible Clindamycin Resistance *Staphylococcus aureus* Clones in Tehran, Iran: A Molecular–Epidemiological Analysis From 2013 to 2018. *Front. Microbiol.* 11, 663. doi: 10.3389/fmicb.2020.00663.

Hait, J. M., Cao, G., Kastanis, G., Yin, L., Pettengill, J. B., and Tallent, S. M. (2021). Evaluation of Virulence Determinants Using Whole-Genome Sequencing and Phenotypic Biofilm Analysis of Outbreak-Linked *Staphylococcus aureus* Isolates. *Front. Microbiol.* 12, 687625. doi: 10.3389/fmicb.2021.687625.

Haque, N., Aung, M. S., Paul, S. K., Bari, M. S., Ahmed, S., Sarkar, S. R., et al. (2019). Molecular Epidemiological Characterization of Methicillin-Susceptible and -Resistant *Staphylococcus aureus* Isolated from Skin and Soft Tissue Infections in Bangladesh. *Microb. Drug Resist.* 25, 241–250. doi: 10.1089/mdr.2018.0123.

He, W.-P., Gu, F.-F., Zhang, J., Li, X.-X., Xiao, S.-Z., Zeng, Q., et al. (2021). Molecular characteristics and risk factor analysis of *Staphylococcus aureus* colonization put insight into CC1 colonization in three nursing homes in Shanghai. *PLOS ONE* 16, e0253858. doi: 10.1371/journal.pone.0253858.

Huang, J., Zhang, F., Zhang, J., Dai, J., Rong, D., Zhao, M., et al. (2021). Molecular Characterization of Rifampicin-Resistant *Staphylococcus aureus* Isolates from Retail Foods in China. *Antibiotics* 10, 1487. doi: 10.3390/antibiotics10121487.

Islam, M. A., Parveen, S., Rahman, M., Huq, M., Nabi, A., Khan, Z. U. M., et al. (2019). Occurrence and Characterization of Methicillin Resistant *Staphylococcus aureus* in Processed Raw Foods and Ready-to-Eat Foods in an Urban Setting of a Developing Country. *Front. Microbiol.* 10, 503. doi: 10.3389/fmicb.2019.00503.

Khan, S., Marasa, B. S., Sung, K., and Nawaz, M. (2021). Genotypic Characterization of Clinical Isolates of *Staphylococcus aureus* from Pakistan. *Pathogens* 10, 918. doi: 10.3390/pathogens10080918.

Kpeli, G., Darko Otchere, I., Lamelas, A., Buultjens, A., Bulach, D., Baines, S. L., et al. (2016). Possible healthcare-associated transmission as a cause of secondary infection and population structure of *Staphylococcus aureus* isolates from two wound treatment centres in Ghana. *New Microbes New Infect.* 13, 92–101. doi: 10.1016/j.nmni.2016.07.001.

Kwapisz, E., Garbacz, K., Kosecka-Strojek, M., Schubert, J., Bania, J., and Międzobrodzki, J. (2020). Presence of egc-positive major clones ST 45, 30 and 22 among methicillin-resistant and methicillin-susceptible oral *Staphylococcus aureus strains*. *Sci. Rep.* 10, 18889. doi: 10.1038/s41598-020-76009-1.

Lebughe, M., Phaku, P., Niemann, S., Mumba, D., Peters, G., Muyembe-Tamfum, J.-J., et al. (2017). The Impact of the *Staphylococcus aureus* Virulome on Infection in a Developing Country: A Cohort Study. *Front. Microbiol.* 8, 1662–1662. doi: 10.3389/FMICB.2017.01662.

Li, X., Huang, T., Xu, K., Li, C., and Li, Y. (2019). Molecular characteristics and virulence gene profiles of *Staphylococcus aureus* isolates in Hainan, China. *BMC Infect. Dis.* 19, 873. doi: 10.1186/s12879-019-4547-5.

Liao, F., Mo, Z., Gu, W., Xu, W., Fu, X., and Zhang, Y. (2020). A comparative genomic analysis between methicillin-resistant *Staphylococcus aureus* strains of hospital acquired and community infections in Yunnan province of China. *BMC Infect. Dis.* 20, 137. doi: 10.1186/s12879-020-4866-6.

Loncaric, I., Lepuschitz, S., Ruppitsch, W., Trstan, A., Andreadis, T., Bouchlis, N., et al. (2019). Increased genetic diversity of methicillin-resistant *Staphylococcus aureus* (MRSA) isolated from companion animals. *Vet. Microbiol.* 235, 118–126. doi: 10.1016/j.vetmic.2019.06.013.

Mairi, A., Touati, A., and Lavigne, J.-P. (2020). Methicillin-Resistant *Staphylococcus aureus* ST80 Clone: A Systematic Review. *Toxins* 12, 119. doi: 10.3390/toxins12020119.

Mairi, A., Touati, A., Pantel, A., Yahiaoui Martinez, A., Ahmim, M., Sotto, A., et al. (2021). First Report of CC5-MRSA-IV-SCCfus “Maltese Clone” in Bat Guano. *Microorganisms* 9, 2264. doi: 10.3390/microorganisms9112264.

Morach, M., Käppeli, N., Hochreutener, M., Johler, S., Julmi, J., Stephan, R., et al. (2019). Microarray based genetic profiling of *Staphylococcus aureus* isolated from abattoir byproducts of pork origin. *PLOS ONE* 14, e0222036. doi: 10.1371/journal.pone.0222036.

Nethercott, C., Mabbett, A. N., Totsika, M., Peters, P., Ortiz, J. C., Nimmo, G. R., et al. (2013). Molecular Characterization of Endocarditis-Associated *Staphylococcus aureus.* *J. Clin. Microbiol.* 51, 2131–2138. doi: 10.1128/JCM.00651-13.

Nhatsave, N., Garrine, M., Messa, A., Massinga, A. J., Cossa, A., Vaz, R., et al. (2021). Molecular Characterization of *Staphylococcus aureus* Isolated from Raw Milk Samples of Dairy Cows in Manhiça District, Southern Mozambique. *Microorganisms* 9, 1684. doi: 10.3390/microorganisms9081684.

Normanno, G., Dambrosio, A., Lorusso, V., Samoilis, G., Di Taranto, P., and Parisi, A. (2015). Methicillin-resistant *Staphylococcus aureus* (MRSA) in slaughtered pigs and abattoir workers in Italy. *Food Microbiol.* 51, 51–56. doi: 10.1016/j.fm.2015.04.007.

Nyasinga, J., Kyany’a, C., Okoth, R., Oundo, V., Matano, D., Wacira, S., et al. (2019). A six-member SNP assay on the iPlex MassARRAY platform provides a rapid and affordable alternative for typing major African *Staphylococcus aureus* types. *Access Microbiol.* 1. doi: 10.1099/acmi.0.000018.

Ohadian Moghadam, S., Modoodi Yaghooti, M., Pourramezan, N., and Pourmand, M. R. (2017). Molecular characterization and antimicrobial susceptibility of the CA-MRSA isolated from healthcare workers, Tehran, Iran. *Microb. Pathog.* 107, 409–412. doi: 10.1016/j.micpath.2017.04.027.

Parisi, A., Caruso, M., Normanno, G., Latorre, L., Sottili, R., Miccolupo, A., et al. (2016). Prevalence, antimicrobial susceptibility and molecular typing of Methicillin-Resistant *Staphylococcus aureus* (MRSA) in bulk tank milk from southern Italy. *Food Microbiol.* 58, 36–42. doi: 10.1016/j.fm.2016.03.004.

Petersen, A., Larssen, K. W., Gran, F. W., Enger, H., Hæggman, S., Mäkitalo, B., et al. (2021). Increasing Incidences and Clonal Diversity of Methicillin-Resistant *Staphylococcus aureus* in the Nordic Countries - Results From the Nordic MRSA Surveillance. *Front. Microbiol.* 12, 668900. doi: 10.3389/fmicb.2021.668900.

Rijnders, M. I. A., Deurenberg, R. H., Boumans, M. L. L., Hoogkamp-Korstanje, J. A. A., Beisser, P. S., and Stobberingh, E. E. (2009). Population Structure of *Staphylococcus aureus* Strains Isolated from Intensive Care Unit Patients in The Netherlands over an 11-Year Period (1996 to 2006). *J. Clin. Microbiol.* 47, 4090–4095. doi: 10.1128/JCM.00820-09.

Sahin-Tóth, J., Kovács, E., Tóthpál, A., Juhász, J., Forró, B., Bányai, K., et al. (2021). Whole genome sequencing of coagulase positive staphylococci from a dog-and-owner screening survey. *PLOS ONE* 16, e0245351. doi: 10.1371/journal.pone.0245351.

Schaumburg, F., Ngoa, U. A., Sters, K. K., Köck, R., Adegnika, A. A., Kremsner, P. G., et al. (2011). Virulence factors and genotypes of *Staphylococcus aureus* from infection and carriage in Gabon. *Clin. Microbiol. Infect.* 17, 1507–1513. doi: 10.1111/j.1469-0691.2011.03534.x.

Schaumburg, F., Pauly, M., Anoh, E., Mossoun, A., Wiersma, L., Schubert, G., et al. (2015). *Staphylococcus aureus* complex from animals and humans in three remote African regions. *Clin. Microbiol. Infect.* 21, 345.e1-345.e8. doi: 10.1016/j.cmi.2014.12.001.

Silva, V., Ribeiro, J., Rocha, J., Manaia, C. M., Silva, A., Pereira, J. E., et al. (2022). High Frequency of the EMRSA-15 Clone (ST22-MRSA-IV) in Hospital Wastewater. *Microorganisms* 10, 147. doi: 10.3390/microorganisms10010147.

Tavares, A., Faria, N. A., Lencastre, H., and Miragaia, M. (2014). Population structure of methicillin-susceptible *Staphylococcus aureus* (MSSA) in Portugal over a 19-year period (1992–2011). *Eur. J. Clin. Microbiol. Infect. Dis.* 33, 423–432. doi: 10.1007/s10096-013-1972-z.

Udo, E. E., Boswihi, S. S., Mathew, B., Noronha, B., Verghese, T., Al-Jemaz, A., et al. (2020). Emergence of Methicillin-Resistant *Staphylococcus aureus* Belonging to Clonal Complex 15 (CC15-MRSA) in Kuwait Hospitals. *Infect. Drug Resist.* Volume 13, 617–626. doi: 10.2147/IDR.S237319.

Vandendriessche, S., De Boeck, H., Deplano, A., Phoba, M.-F., Lunguya, O., Falay, D., et al. (2017). Characterisation of *Staphylococcus aureus* isolates from bloodstream infections, Democratic Republic of the Congo. *Eur. J. Clin. Microbiol. Infect. Dis.* 36, 1163–1171. doi: 10.1007/s10096-017-2904-0.

Vubil, D., Garrine, M., Ruffing, U., Acácio, S., Sigaúque, B., Alonso, P. L., et al. (2017). Molecular Characterization of Community Acquired *Staphylococcus aureus* Bacteremia in Young Children in Southern Mozambique, 2001-2009. *Front. Microbiol.* 8, 730–730. doi: 10.3389/fmicb.2017.00730.

Wang, X., Zhao, H., Wang, B., Zhou, Y., Xu, Y., Rao, L., et al. (2022). Identification of methicillin-resistant *Staphylococcus aureus* ST8 isolates in China with potential high virulence. *Emerg. Microbes Infect.* 11, 507–518. doi: 10.1080/22221751.2022.2031310.
