# Supplementary material for: Antimicrobial resistance and clonality of Staphylococcus aureus causing bacteraemia in children admitted to the Manhiça District Hospital, Mozambique, over two decades
Source: Front Microbiol. 2023 Jul 24;14:1208131. doi: 10.3389/fmicb.2023.1208131 (PMC10406509; doi:10.3389/fmicb.2023.1208131)
Supplement: Supplementary file 3 [file Table_3.doc]

Supplementary Material

**Title:** Antimicrobial resistance and clonality of *Staphylococcus aureus* causing bacteraemia in children admitted to the Manhiça District Hospital, Mozambique, over two decades

**Authors:** Marcelino Garrine^1,2^, Sofia Santos Costa^2^, Augusto Messa Jr^1^, Sérgio Massora^1^, Delfino Vubil^1^, Sozinho Ácacio^1,3^, Tacilta Nhampossa^1,3^, Quique Bassat^1,4,5,6,7^, Inácio Mandomando^1,3,4^ and Isabel Couto^2*^

***Correspondence:**Isabel Couto

Email: [icouto@ihmt.unl.pt](mailto:icouto@ihmt.unl.pt)

**Table S3. Resistance profiles among multidrug resistant (MDR) and methicillin resistant**

**(MRSA) *S. aureus* (N = 86).**

| **Resistance patterns profile** | **MDR % (n)** | **MRSA % (n)** |
| --- | --- | --- |
| FOX-TCY-PEN | 0 | 1 (1) |
| ERY-CLID-TCY | 1 (1) | 0 |
| CHL-ERY-CLID-PEN | 1 (1) | 0 |
| CHL-ERY-CLID-TCY-PEN | 1 (1) | 0 |
| CHL-TCY-PEN | 1 (1) | 0 |
| ERY-TCY-PEN | 1 (1) | 0 |
| GEN-SXT-CIP-PEN | 1 (1) | 0 |
| SXT-CHL-ERY-CLID-TCY-PEN | 1 (1) | 0 |
| SXT-CHL-TCY-PEN | 1 (1) | 0 |
| FOX-ERY-CLID-PEN | 1 (1) | 1 (1) |
| FOX-ERY-CLID-TCY-PEN | 1 (1) | 1 (1) |
| GEN-SXT-FOX-ERY-CLID-TCY-PEN | 1 (1) | 1 (1) |
| SXT-CHL-FOX-ERY-CLID-TCY-PEN | 1 (1) | 1 (1) |
| GEN-FOX-ERY-CLID-TCY-PEN | 2 (2) | 2 (2) |
| SXT-ERY-CLID-PEN | 7 (6) | 0 |
| SXT-ERY-CLID-TCY-PEN | 7 (6) | 0 |
| SXT-TCY-PEN | 8 (7) | 0 |
| GEN-SXT-CHL-FOX-ERY-CLID-TCY-PEN | 10 (9) | 10 (9) |
| ERY-CLID-PEN | 20 (17) | 0 |
| ERY-CLID-TCY-PEN | 30 (26) | 0 |

PEN, penicillin; FOX, cefoxitin; TCY, tetracycline; ERY, erythromycin; CLI, clindamycin; SXT, co-trimoxazole; CHL, chloramphenicol; GEN, gentamicin.
