# Supplementary material for: Antimicrobial resistance and clonality of Staphylococcus aureus causing bacteraemia in children admitted to the Manhiça District Hospital, Mozambique, over two decades
Source: Front Microbiol. 2023 Jul 24;14:1208131. doi: 10.3389/fmicb.2023.1208131 (PMC10406509; doi:10.3389/fmicb.2023.1208131)
Supplement: Supplementary file 4 [file Table_4.doc]

Supplementary Material

**Title:** Antimicrobial resistance and clonality of *Staphylococcus aureus* causing bacteraemia in children admitted to the Manhiça District Hospital, Mozambique, over two decades

**Authors:** Marcelino Garrine^1,2^, Sofia Santos Costa^2^, Augusto Messa Jr^1^, Sérgio Massora^1^, Delfino Vubil^1^, Sozinho Ácacio^1,3^, Tacilta Nhampossa^1,3^, Quique Bassat^1,4,5,6,7^, Inácio Mandomando^1,3,4^ and Isabel Couto^2*^

***Correspondence:**Isabel Couto

Email: [icouto@ihmt.unl.pt](mailto:icouto@ihmt.unl.pt)

**Table S4. Frequency and level of agreement between antibiotic susceptibility**

**testing (AST) and its resistance determinants**

| **Penicillin (PEN)** | | | |
| --- | --- | --- | --- |
| Antibiotic susceptibility testing | Resistance determinant: *blaZ* | | Total |
|  | Negative | Positive |  |
| Susceptible | 31 | 1 | 32 |
| Resistant | 1 | 303 | 304 |
| Total | 32 | 304 | 336 |
| Agreement (%) | 99.40% |  |  |
| Kappa coefficient | 0.965 | 95% CI: 0.918 to 1.000 |  |
| **Cefoxitin (FOX)** | | | |
| Antibiotic susceptibility testing | Resistance determinant: *mecA* | | Total |
|  | Negative | Positive |  |
| Susceptible | 320 | 0 | 320 |
| Resistant | 0 | 16 | 16 |
| Total | 320 | 16 | 336 |
| Agreement (%) | 100% |  |  |
| Kappa coefficient | 1.000 | 95% CI: 1.000 to 1.000 |  |
| **Tetracycline (TCY)** | | | |
| Antibiotic susceptibility testing | Resistance determinant: *tet(*K) | | Total |
|  | Negative | Positive |  |
| Susceptible | 166 | 9 | 175 |
| Resistant | 39 | 122 | 161 |
| Total | 205 | 131 | 366 |
| Agreement (%) | 85.71% |  |  |
| Kappa coefficient | 0.716 | 95% CI: 0.637 to 0.786 |  |
