# Supplementary material for: Antimicrobial resistance and clonality of Staphylococcus aureus causing bacteraemia in children admitted to the Manhiça District Hospital, Mozambique, over two decades
Source: Front Microbiol. 2023 Jul 24;14:1208131. doi: 10.3389/fmicb.2023.1208131 (PMC10406509; doi:10.3389/fmicb.2023.1208131)
Supplement: Supplementary file 5 [file Table_5.doc]

Supplementary Material

**Title:** Antimicrobial resistance and clonality of *Staphylococcus aureus* causing bacteraemia in children admitted to the Manhiça District Hospital, Mozambique, over two decades

**Authors:** Marcelino Garrine^1,2^, Sofia Santos Costa^2^, Augusto Messa Jr^1^, Sérgio Massora^1^, Delfino Vubil^1^, Sozinho Ácacio^1,3^, Tacilta Nhampossa^1,3^, Quique Bassat^1,4,5,6,7^, Inácio Mandomando^1,3,4^ and Isabel Couto^2*^

***Correspondence:**Isabel Couto

Email: [icouto@ihmt.unl.pt](mailto:icouto@ihmt.unl.pt)

**Table S5. Phenotypic resistance and resistance determinants among all *S. aureus* clonal complexes detected in this study.**

| **CC** | **Resistance patterns (n)** | **Main resistance determinants (n)** |
| --- | --- | --- |
| **CC5** (N=58,  MRSA: 0;  MDR: 6 (10%)) | PEN (31) | *blaZ* (31) |
|  | PEN-TCY (14) | *blaZ*-*tet*(K) (12); *blaZ-tet*(K)*-tet*(L)*-tet*(M) (1); *blaZ-tet*(L)*-tet*(M) (1) |
|  | PEN-TCY-ERY-CLID (2) | *blaZ-tet*(K)*-erm*(C) (1); *blaZ-tet*(L)-*tet*(M)*-msr*(A) (1) |
|  | PEN-ERY-CLID (2) | *blaZ* (1); *blaZ-erm*(C) (1) |
|  | PEN-SXT-ERY-CLID (1) | *blaZ-tet*(K)*-dfrG* (1) |
|  | TCY-ERY-CLID (1) | *tet*(K) (1) |
|  | PEN-ERY (1) | *blaZ-erm*(C) (1) |
|  | ERY-CLID (1) | *erm*(C) (1) |
|  | Fully susceptible (5) | *blaZ* (1) |
| **CC8** (N=56,  MRSA: 15 (27%);  MDR: 22 (39%))  CC8 (N=56) | PEN (15) | *blaZ* (14); *blaZ-tet*(K) (1) |
|  | PEN-TCY (16) | *blaZ*-*tet*(K) (13); *blaZ*-*tet*(K)-*tet*(L) (1); *blaZ*-*tet*(L)-*tet*(M) (1); *blaZ*-*tet*(L) (1) |
|  | FOX-PEN-TCY-GEN-SXT-CHL-ERY-CLID (9) | *blaZ-tet*(M)*-mecA-erm*(C)*-aacA_aphD-dfrA*(S1*)-cat* (5); *blaZ-tet*(K)*-tet*(M)*-mecA-erm*(C)*-msrA-aacA_aphD-dfrA*(S1)*-dfrG-cat* (1); *blaZ-tet*(L)*-tet*(M)*-mecA-erm*(C)*-aacA_aphD-dfrA*(S1) (1); *blaZ-tetM-mecA-ermC-aacA_aphD-dfrA*(S1) (1); *blaZ-tet*(K)*-tet*(M)*-mecA-erm*(C)*-aacA_aphD-dfrA*(S1) (1) |
|  | PEN-TCY-ERY-CLID (5) | *blaZ-tet*(L)*-tet*(M)*-erm*(C) (3); *blaZ-tet*(K)*-erm*(C) (1); *blaZ* (1) |
|  | FOX-PEN-TCY-GEN-ERY-CLID (2) | *blaZ-tet*(K)*-tet*(L)*-tet*(M)*-mecA-erm*(C)*-aacA_aphD* (1); *blaZ-tet*(L)*-tet*(M)*-mecA-erm*(C)*-aacA_aphD* (1) |
|  | FOX-PEN-TCY-GEN-SXT-ERY-CLID (1) | *blaZ-tet*(K)*-tet*(M)*-mecA-erm*(C)*-msrA-aacA_aphD-dfrA*(S1) (1) |
|  | FOX-PEN-TCY-SXT-CHL-ERY-CLID (1) | *blaZ-tet*(M)*-mecA-erm*(C)*-dfrA*(S1)*-cat* (1) |
|  | FOX-PEN-TCY-ERY-CLID (1) | *mecA-blaZ-tet*(L)*-tet*(M)*-erm*(C) (1) |
|  | FOX-PEN-TCY (1) | *mecA-blaZ-tet*(K)*-tet*(L)*-tet*(M) (1) |
|  | PEN-TCY-SXT (2) | *blaZ-tet*(K)*-dfrG* (2)  *,* |
|  | PEN-ERY (1) | *blaZ* (1) |
|  | PEN-TCY-CHL-ERY-CLID (1) | *blaZ-tet*(L)*-erm*(C) (1) |
|  | PEN-CHL (1) | *blaZ* (1) |

**Table S5. *(Cont.)* Phenotypic resistance and resistance determinants among all *S. aureus* clonal complexes detected in this study.**

| **CC** | **Resistance patterns (n)** | **Main resistance determinants (n)** |
| --- | --- | --- |
| **CC15** (N=37,  MRSA:0,  MDR: 2 (5%)) | PEN (18) | *blaZ* (16); *blaZ-tet*(K) (1); *blaZ-tet*(K)*-tet*(L) (1) |
|  | PEN-TCY (17) | *blaZ*-*tet*(K) (16); *blaZ-tet*(K)*-tet*(L) (1) |
|  | ERY-CLID-TCY-PEN (2) | *blaZ-tet*(K)*-erm*(C) (2) |
| **CC1** (N=36,  MRSA: 0;  MDR: 9 (25%)) | PEN (14) | *blaZ* (12); *blaZ-tetK* (2) |
|  | PEN-TCY (7) | *blaZ*-*tet*(K) (5); *blaZ-tet*(K)*-tet*(L) (1); *blaZ* (1) |
|  | PEN-TCY-ERY-CLID (3) | *blaZ*-*tet*(K)-*erm*(C)-*msr*(A) (1); *blaZ*-*tet*(K)-*erm*(C) (1); *blaZ*-*tet*(L)- *tet*(M) (1) |
|  | PEN-ERY-CLID (3) | *blaZ*-*erm*(C) (3) |
|  | PEN-TCY-SXT-ERY-CLID (1) | *blaZ-tet*(K)*-erm*(C) (1) |
|  | PEN-TCY-SXT (1) | *blaZ-tet*(K)*-dfrG* (1) |
|  | PEN-TCY-CHL (1) | *blaZ-tet*(K)*-cat* (1) |
|  | PEN-ERY (1) | *blaZ* (1) |
|  | PEN-CLID (1) | *blaZ* (1) |
|  | TCY (2) | *tet*(M) (1) |
|  | Fully susceptible (2) |  |
| **CC121** (N=34,  MRSA: 0;  MDR: 9 (26%)) | PEN-TCY (11) | *blaZ-tet*(M) (7); *blaZ-tet*(K) (2); *blaZ-tet*(K)-*tet*(L) (1); *blaZ* (1) |
|  | PEN (10) | *blaZ* (10) |
|  | PEN-TCY-ERY-CLID (6) | *blaZ*-*tet*(K)-*erm*(C) (6) |
|  | PEN-ERY (3) | *blaZ-erm*(C) (2); *blaZ* (1) |
|  | PEN-SXT-ERY-CLID (1) | *blaZ- dfrG-ermC* (1) |
|  | PEN-SXT-TCY (1) | *blaZ- dfrG-tetM* (1) |
|  | PEN-ERY-CLID (1) | *blaZ-erm(C)* (1) |
|  | Fully susceptible (1) |  |
| **CC152** (N=33,  MRSA: 0;  MDR: 12 (36%)) | PEN (14) | *blaZ* (14)  *blaZ-tet*(K)-*tet*(L) (3)  *blaZ-tet*(K)-*erm*(C) (2) |
|  | PEN-ERY-CLID (8) | *blaZ* (3); *blaZ-erm*(C) (3); *blaZ-tet*(K)*-erm*(C)*-msr*(A) (1); *blaZ-tet*(K)*-erm*(C) (1) |
|  | PEN-TCY (5) | *blaZ-tet*(K)*-tet*(L) (3); *blaZ-tet*(M)*-tet*(L) (1); *blaZ-tet*(K) (1) |
|  | PEN-TCY-ERY-CLID (4) | *blaZ-tet*(K)*-tet*(L)*-erm*(C) (1); *blaZ-tet*(K)*-erm*(C) (1); *blaZ-tet*(M)*-erm*(C) (1); *blaZ-erm*(C) (1) |
|  | Fully susceptible (2) |  |
| **CC88** (N=26,  MRSA: 1 (4%); MDR: 6 (23%)) | PEN-TCY (15) | *blaZ-tet*(K) (14); *blaZ-tet*(L)-*tet*(M) (1) |
|  | PEN (2) | *blaZ* (2) |
|  | PEN-ERY (2) | *blaZ* (2) |
|  | PEN-TCY-ERY-CLID (2) | *blaZ-tet*(L)*-tet*(M)*-erm*(C) (1); *blaZ-tet*(K) (1) |
|  | FOX-PEN-ERY-CLID (1) | *blaZ-mecA-erm*(C) (1) |
|  | PEN-SXT-ERY-CLID (1) | *blaZ-erm*(C) (1) |
|  | PEN-TCY-ERY (1) | *blaZ-tet*(K) (1) |
|  | PEN-ERY-CLID (1) | *blaZ-erm*(C) (1) |
|  | PEN-SXT (1) | *blaZ* (1) |

**Table S5. *(Cont.)* Phenotypic resistance and resistance determinants among all *S. aureus* clonal complexes detected in this study.**

| **CC** | **Resistance patterns (n)** | **Main resistance determinants (n)** |
| --- | --- | --- |
| **CC25** (N=20,  MRSA: 0;  MDR: 16 (80%)) | PEN-TCY-SXT-ERY-CLID (5) | *blaZ-tet*(K)*-erm*(C)*-dfrG* (5) |
|  | PEN-SXT-ERY-CLID (3) | *blaZ-erm(C)-dfrG* (); *blaZ-erm(C)* (1) |
|  | PEN-TCY-SXT (3) | *blaZ-tet*(K)*-dfrG* (3) |
|  | PEN-SXT (2) | *blaZ-dfrG* (1); *blaZ* (1) |
|  | PEN-TCY-SXT-CHL-ERY-CLID (1) | *blaZ-tet*(K)*-erm*(C)-*dfrG* (1) |
|  | PEN-TCY-SXT-CHL (1) | *blaZ-tet*(K)*-dfrG-cat* (1) |
|  | PEN-TCY-ERY-CLID (1) | *blaZ-tetK-erm*(C) (1) |
|  | PEN-CHL-ERY-CLID (1) | *blaZ-erm(C)-cat* (1) |
|  | PEN-ERY-CLID (1) | *blaZ-msrA* (1) |
|  | PEN-TCY (1) | *blaZ-tet*(K) (1) |
|  | PEN (1) | *blaZ* (1) |
| **CC80**  (N=16,  MRSA: 0;  MDR: 1 (6%)) | TCY (7) | *tet*(K) (7) |
|  | PEN-ERY-CLID (1) | *blaZ-erm*(C) (1) |
|  | PEN-TCY (1) | *blaZ-tet*(K) (1) |
|  | Fully susceptible (7) |  |
| **CC45**  (N=8,  MRSA: 0,  MDR: 1 (13%)) | PEN (4) | *blaZ* (4) |
|  | PEN-TCY (2) | *blaZ-tet*(K) (2) |
|  | PEN-TCY-ERY-CLID (1) | *blaZ*-*tet*(K)-*erm*(C) (1) |
|  | Fully susceptible (1) |  |
| **CC22**  (N=6,  MRSA: 0,  MDR: 1 (17%)) | PEN (3) | *blaZ* (3) |
|  | PEN-GEN (2) | *blaZ-aacA_aphD* (2) |
|  | PEN-GEN-SXT-CIP (1) | *blaZ-dfrA(S1)* (1) |
| **CC12**  (N=3,  MRSA: 0,  MDR: 3 (100%)) | PEN (2) | *blaZ* (1); *blaZ-tet*(K) (1) |
|  | TCY (1) | *tet*(K) (1) |
| **Singleton**  (N=2,  MRSA: 0,  MDR: 0) | PEN-TCY (2) | *blaZ-tet*(K) (2) |
|  | TCY-ERY (1) | *tet(*K) (1) |
| **CC97**  (N=1,  MRSA: 0,  MDR: 0) | Fully susceptible (1) |  |

PEN, penicillin; FOX, cefoxitin; TCY, tetracycline; ERY, erythromycin; CLID, clindamycin; SXT, co-trimoxazole; CHL, chloramphenicol; GEN, gentamicin; CIP, ciprofloxacin.
